# Supplementary material for: Developing a hope-focused intervention to prevent mental health problems and improve social outcomes for young women who are not in education, employment, or training (NEET): A qualitative co-design study in deprived coastal communities in South-East England
Source: PLoS One. 2024 May 31;19(5):e0304470. doi: 10.1371/journal.pone.0304470 (PMC11142577; doi:10.1371/journal.pone.0304470)
Supplement: S1 Table — Illustrative quotes are presented, labelled with participant number. (DOCX) [file pone.0304470.s001.docx]

**S1 Table. Data analysis of Phase 1 stakeholder research interviews conducted with NEET young women, family members, and health and community practitioners.** Illustrative quotes are presented, labelled with participant number.

| **Research Question** | **TiDiER item** | **Sub-theme** | **Sub-theme description** | | |
| --- | --- | --- | --- | --- | --- |
|  |  |  | **Young women** | **Relatives** | **Professional** |
| 1: What content areas, format(s) and setting(s) are viable for delivering a brief, low-cost, hope-focused intervention to NEET young women aged 16-24 years in deprived coastal communities? | WHY | Rationale for psychological intervention explicitly focused on enhancing hope | NEET young women agreed that a hopeful intervention designed specifically for them sounded “*really good*” (S10) and “*really interesting*” (K9), emphasising its novelty and relevance;  “…*obviously there’s like support out there, like mental health, but I’ve never really heard of anything like this, so it would definitely be useful*” (S13)  “…*very helpful to a lot of people… there’s a lot of people struggling in that sense*” (K9)  “*I think it’s an amazing idea…I was really happy…it was like, “Finally someone realises” that…it’s becoming a bigger issue, …hope for the future, so that hopefully, one day, less young women and girls have to go through what I went through, that’s a big thing*” (S16).  NEET young women were identified as a group that were unfairly maligned and unsupported, making a hope-focused intervention particularly relevant:  “*…people wouldn’t feel that they’re so much of a burden to people, or they’re such a disgrace to society…young women are the most…misunderstood…normally is the age bracket where we’re going out partying, getting drunk, and because we’re not doing that, it’s like one of the most hidden disabilities…I think if that help’s there to ease a lot of stuff for a lot of young women and make it a lot more comfortable and easier for them to want to go and find employment or education or training, because they can see that the helps there for them*” (K7). | Parents agreed that a hopeful intervention is highly relevant to NEET young women; “*I think it’s a really good idea, I think anything that gives them the opportunity, I think that’s a big thing*” (S8).  A hopeful intervention was seen to have the potential to first fill a gap in helping NEET young women to clarify their aspirations:  “*I don’t think there is anything there for young people who aren’t in education, and they can’t go straight into a job because they’ve had no experience, there has to be something there for people to…focus on…something for them to say, “Okay right, I could go here and they could help me*”” (K11).  Secondly, a hopeful intervention was deemed able to imbue young women with confidence linked to such aspirations:  “… *giving them confidence to actually go out there and say “Yes, I am an adult now and you know I want to do something with my life, I don’t want to just be labelled as a drop-out or you know, I actually want to do something with my life.” And I think a lot of young women may feel like that but feel that there’s nothing there to support them in order to do that…obviously you gain confidence from you know knowing a little bit about what you’re doing*” (K11). | Practitioners espoused the relevance of a hope-focused intervention for NEET young women in coastal communities. Hope was considered a universal factor that could benefit anyone:  “*I don't think you can understate* [hope’s] *importance because no matter what the person’s social or mental health*” (K4; non-NHS)  “*It might be a really different sort of approach because it’s independent of all the stuff I’ve been talking about, you know, the difficulties, because hope, hope, it hopefully transcends that, doesn’t it? If you have hope that you can move forward, that you can perhaps have a better life than you thought you were going to, or you might be, or to even envisage that, to think about it and hope to get towards it, I think it could be a fantastic thing… not feeling that young people are, that this isn’t going to make your depression go away, it isn’t going to get you housed, but it might do in a way …it depends how far you take it…it would be a lovely thing to have if they’re a regular thing as well, “What are we doing? We’re just being hopeful.”*” (S4; non-NHS).  However, hope was considered of particular importance to NEET young women, who were considered to be especially low in hope and influenced by intergenerational and broader sociocultural transmission of pessimism:  “*I think it’s relevant, I think we need to raise not necessarily educational ambition but their self-belief, and their “Yeah, I can do this, I can have a go at this, and actually it’s okay to fail”*" (S5; non-NHS).  “…*not looking at moving from NEET into EET as a negative thing which sometimes can… they can be told that quite a lot, you know, especially if they're from intergenerational households where… they don't work. Going to work as a bad thing in terms of, “Why do you want to do that? Why do you want to work for somebody?”*” (S2; non-NHS).  “*I’m talking about the girls that I see down here and the lack of respect they get…that’s a fundamental part of girls being not aspirational sometimes…Girls in here, really good smashing girls, and as soon as they can they want to work…*[but] *more than half these lads that they get involved with that say to them, “Oh you know we’ll have a family of our own”…and it’s music to some people's ears when they haven’t experienced that love and have someone taking an interest in them…And people look down on girls that have babies at a young age and stuff…one particular girl I’m seeing this afternoon,…I remember her saying,” Oh I’m going to go back to college, if I can, I want my daughter to see me working, I don’t want to be like this.” And it’s quite hard for them to sort of do that, especially when they’ve got anxiety, depression involved*.” (S4; non-NHS).  Hope was seen to be fundamental to positive change:  “…*that's when you really start to see a shift in their wellbeing…if they feel that hope in one route to achieving something then that’s quite powerful*” (S3, NHS)  “*And targeting just that, that area, because it could be what just lifts, what makes the difference to some young people*” (S4; non-NHS).  Intervening in hope to scaffold engagement in meaningful activity was avidly encouraged to interrupt the potential long-term impacts of a period of being NEET:  “…*if these young people don't get some reasonably intensive support to get back into meaningful activities where they feel some joy at least in what they're doing and therefore feel valued and feel that they're contributing something, feel that they have a reason to be there, …if you follow their trajectories into their late 20s early 30s, they go completely off the radar and we lose them completely from our system*” (S11; NHS).  Hope was additionally considered a novel intervention approach, “*it’s a very good concept but I haven’t come across any existing currently… locally”* (K1; non-NHS), that had the potential to be more attractive and less stigmatising than problem-focused interventions:  “*I’d pitch it to people as being like having a personal trainer. If you took a sort of like, this is almost like getting fit, but you're getting life-fit you know, anything that de-stigmatises and makes it about you taking control and then being your best self*” (S19; NHS)  “…*I mean community health worker and mental health nurses, they do identify but they don’t have, I mean beyond that the main aim of them is to treat the disease*” (K1; non-NHS).  Practitioners emphasised that the intervention, therefore, should explicitly focus on hope; “*The approach has to be hopefulness-based because otherwise, why bother?*” (S11; NHS). Practitioners additionally emphasised that being involved in delivering a hopeful intervention would be beneficial for providers; “*…our staff would really love to be involved in delivering some information about that hopefulness …that would be really a buoyant thing to do and for everyone to be part of*” (S9; non-NHS). |
|  | WHAT |  |  |  |  |
|  | Materials | Participant workbook available online/paper-based | Some young people recommended an online workbook to support intervention delivery, “…*online because it’s easy to access and quite a lot of things are online now*” (S10), and some advised paper-based; “...*actual paper. I mean a lot of stuff is done on the computer and stuff, which is okay, but I’m more of a visual person, like right in front of me, I can hold it*” (S13). The option for either online or on paper appeared to be important way to account for these individual preferences: “…*send to me on an email but then print of a sheet as well so I have both*” (S13). |  | Individual practitioners reported that young people would most prefer an online workbook; “…*most of them want to do online, I think most of them don't really want to be handwriting*” (S2; non-NHS). However, paper-based materials offered potential benefits as a ‘transitional object’ in between sessions and an aide-memoir after the intervention had been completed:  “…*something that is tangible so that perhaps the person does leave with a postcard or a something or a link to whatever it is, but they need something. Something then they can remind themselves between then and the next appointment that actually, you know that there is hope. Because I think people need that kind of object to hold on to as well to remind them of the message, but like I said… if you enter into that kind of transaction, you have to, you know you’re duty-bound, certainly as a professional, but also as a human being to follow up on it and make sure that if you are going to promote hope for someone, that you don't then dash it all by just forgetting about it*” (S11; NHS)  “…*print any resources they feel that they need… it's nice to have things to look back on and perhaps having that as a hard copy would benefit some*” (S1; non-NHS).  Therefore, offering both paper and online materials appeared to be the best solution to meeting young people’s differing needs; “…*we're doing a kind of mix, both, at the moment. Some kind of paper and pen, and some are online…that's something that you can ask at the time as a preference*” (S12; NHS). |
|  | Procedures | Inclusive entry during late adolescent/ early adulthood early intervention staging, with focus on offering before/during transition points | The optimum time to deliver an intervention was considered to be during the late adolescent/early adulthood period;  “…*late teenagers, early adulthood*” (K7)  “*I think younger people who are struggling with mental health and maybe like people who are still in school by maybe like probably around like 16 to like 20, that kind of age group*” (K9).  This age period was emphasised as a time in which the intervention would be particularly relevant and helpful; for young people were experiencing transitions in which they were particularly vulnerable:  “…*it’s a big jump from leaving school to going to university or something like, it’s a big time, it’s a big change as well I feel moving into your first home as a young person like can be quite stressful and quite overwhelming for you and it could have quite a bad impact on you especially if you’re like sitting exams or going through college or university, I feel like that age range could benefit the most*” (K7)  Additional examples of challenging transitions included leaving compulsory education, and becoming a parent or carer. Despite such challenges, young women were identified as having particular problems accessing appropriate (or any) support; “*A lot of people my age…a lot of the times they struggle to get professional help*” (K9). A hopeful intervention could be offered as a form of early intervention when a person first shows signs of stress and problems coping:  “…*as soon as they start struggling, whether that be with like their mental health or their home situation or education. I think that due to just the total lack of support now, it would be better if it was done as soon as there’s sort of sign of struggle. Because otherwise it might be really severe and it’s a lot harder to come back from that*” (S16).  However, it was additionally recommended to be inclusive and avoid instituting a strict cut-off point with respect to age:  “…*and also* [avoid] *a cut-off point for like, there are a lot of the services that as soon as you turn 18, “We can’t support you” and so it was like rushing to get that support. So even if I’d been in contact with them before, to know that I had a complete cut-off point as opposed to them supporting me for a little bit after that, I think that would put me off*” (S16).  This was considered especially the case at the upper age limit in the context of reduced support opportunities for older adolescents:  “…*a lot of the services that do offer support is young teenagers and as someone who’s 20, I still need that support but there’s not very many services that offer that*” (S16). | Parents agreed that a hopeful intervention should be offered during the late adolescent/early adulthood period in the context of the transition to adult occupation:  “…*really difficult to go from kind of not so much responsibility to being like “Oh, my god, you know, I’ve got to do this now, and where do I go, what do I do?”, you know I think there’s a gap between them leaving school and then deciding what to do with the rest of your life*” (K11).  There were varying opinions on whether before or just after leaving compulsory education might be best:  “*I think maybe potentially before these young people leave school because not everyone goes to you know to the end of term, which is what* [daughter] *did, she dropped out maybe the schools might have access this to something like what you said, so they can explain to people, young people before they leave, that there is something out there, there is, like you say, “There’s hope”*” (S11).  Early intervention was considered important, beginning as soon as possible after people became NEET or at risk thereof:  “*I think it has to happen quite quick, because I think if people are kind of left to stew on their own, you know they kind of get out of sync of, “Oh I don’t want to get up today, I’ve got nothing to get up for.”*” (K11). | Practitioners emphasised the broad and transdiagnostic relevance and likely benefits of a hope-focused intervention, “*I think everyone can benefit from it…* *whether they come from backgrounds of trauma, whether it's substance use, whether it's neurodiversity, whether it's depression, anxiety, eating disorder, all of the different kind of presentations we have*” (S11; NHS). Practitioners identified the late adolescence to early adulthood as the best period in which to deliver a hopeful intervention; “*These are the crucial years in a young adult’s or older adolescent’s* [life]*…the 15 to 25 age range that I work with*” (S11; NHS). Practitioners situated the delivery of a hopeful intervention as appropriate for ‘early intervention’, for example suggesting it could be before leaving school in order to raise aspiration and engagement. However, practitioners additionally stated that the intervention felt appropriate for delivery once young women were NEET, for example, provided by support services if young women were in contact with these:  “*I would suggest it would be best launched at the beginning of their journey. But to have it have open, and open parameters, so that once the person - I think once the person is on board with that service, however it is configured in the future, I think the important thing is that the person can come at the start of their journey, and then stay and receive a service from the hopefulness project, until they choose, that they feel they've had the launchpad, it's almost like a springboard to achieve their goals*” (K4; non-NHS).  One professional emphasised that delivering the intervention to young women who were long-term NEET may be more challenging:  “*If you're recently out of employment or training or education, it's not been a couple of years or three years, then I think it's easier to be hopeful. If you've struggled for three years to turn things around and they're not turning around, it’s more difficult then to meaningfully say “Don't give up hope”, because someone might say “Well it’s alright for you to say that but I've been stuck in this place for three years”*” (S11; NHS). |
|  |  | Accessible information about the intervention in multiple forms, targeting multiple stakeholders | Young people emphasised the importance of increasing the probability that they would be informed about a hopeful intervention, emphasising the challenge of of knowing what support options are available to them:  “*…you never really see anything kind of advertised or anything that you know specifically to go to…you don’t really know how to reach it and it just kind of, I don’t know…there should definitely be more recognition, like advertising, just so that people know where to find it, because some people will just, won’t know where to start*” (S10).  In particular, better online information about support options was needed; “…*it’s just helpful to be aware of these things, like sometimes it’s quite difficult to find them online*” (S6). However, printed materials potentially function better as an aide-memoir “…*it’s very helpful to have information or if someone mentioned it, I would like forget the name when I got home, so it was very nice to have it on like a piece of paper with all of the information*” (S10). | Parents emphasised the challenge of understanding what support services are available for NEET young women:  “*I wish there was something that they could go to, but, there kind of is but you have to access it yourself. You know you’re not given that information straight away…* [daughter] *only found out from us going to see these people at the job centre and them forwarding us onto other people and organisations*” (K11).  Parents described supporting their children to access support, by researching options using the internet; “*I basically went on the internet and looked for private counsellors … so me and* [daughter] *went through them all together*” (K11). | Practitioners argued that informational materials for interventional support should be informed by NEET young women to ensure they are appropriate. Moreover, online promotion needs to be strategic and target the online spaces NEET women actually inhabit:  “*Some of the materials and the way they’re promoted can be the first hurdles…You need to make sure that the material you're putting out there is eye-catching… because that's the first way to try and engage with* *them…some of the basics are good social media promotion, a nice, bold flyer with an appropriate font, a good marketing team, even perhaps getting a little focus group*. *Sometimes things are designed by people who are so far removed from a NEET young person…and it needs to be done in the right channels, so obviously social media, TikTok particularly…just any carrots and broadening horizons*” (S2; non-NHS).  Practitioners reported that help-seeking was often supported by family members: “…*for the younger ones, they might still be at home, so parents will access help*” (S11; NHS). Therefore, targeting family members was encouraged as a route to try and engage isolated NEET young women:  “...*parents and grandparents for the hidden NEETs, so, perhaps getting promotional material and the messages through their groups because that's where you'll find the hidden NEETs, otherwise you won't find them. The hidden NEETs that we get referred were generally by family members that would see a flyer or hear about it, otherwise you won't hear from them*” (S2; non-NHS). |
|  |  | Supported intervention delivery | Young women emphasised that having a provider support the intervention delivery was beneficial, and itself hope-inspiring:  “*I think feeling supported is a big part of it and having other people hold the hope for you when you can’t hold it yourself…to receive support shows that somebody cares and believes in you. I think yeah to have someone helping you and guiding you is quite comforting and yeah, I’d say that really helps to develop hopefulness”* (S7)  Interpersonal support was considered fundamental to enhancing hope; “*I think that I wouldn’t be able to feel hopeful without getting support*” (K9). In particular, a non-changing supporter was wanted:  “…*I think more consistency in support*” (S7)  “…*once I’ve talked to someone and that, I can’t really, it’s hard to thingy up with someone else*” (K8). | Parents emphasised the importance of a hopeful intervention occurring in the context of an interpersonal relationship, because a human ‘face’ to interventions itself contributes to enhancing hope:  “*…to think that somebody outside is prepared to put that sort of investment into you, that hope, would be quite important I should think for any young girl to think that somebody out there who actually does think that they’re worth it and they’re worth, you know, doing more*” (S8). | Practitioners emphasised the importance of the hopeful intervention being provided within a relational context, rather than a purely self-directed package; “*I wouldn't want to do an online module on hope. It would feel really kind of disconnected and meaningless*“ (S11; NHS). Having a supporter for individual, and any group, intervention components was considered best and safest practice, “…*it's good to have a professional there to answer any questions and…safeguarding and stuff like that*” (S18; non-NHS). Moreover, a supporter was considered one of, if not the most, powerful vehicles for enhancing hope in itself, especially in the context of the likelihood that NEET young women had experienced adversity and lacked access to positive relationships:  “…*one of the most powerful things when we promote hope is to put forward the idea that actually “I’m invested in this as well and I’m doing this because I care and I believe things will get better for you. I'm not just saying that because I’m paid to do it because I believe it”, and if you can get that message across, I think that's incredibly powerful…I think hope is something that you have to kind of foster and then nurture and then support… for women who are NEET at a young age, you can pretty much guarantee that they've not had a great life up until that point…they're not going to be in a great place*” (S11; NHS)  “…*supported, just because the group would probably feel more supported…and that might give them more hope that someone's with them on that journey*” (S12; non-NHS). |
|  |  | Gentle start to intervention | Young people encouraged a gentle introduction to the intervention; “…*like I said, early* [on], *taking things one step at a time*” (K9). This was seen as necessary with respect to meeting the provider and in terms of the intervention content and focus. One participant suggesting a pre-intervention meeting with the supporter; “…*meeting beforehand with like the person so then you get to like introduce them and then when you go back you find more comfortable*” (S10). Other participants emphasised the need to take the introduction to setting future goals slowly, for example:  “…*early kind of taking things one step at a time, because I know life and the future and jobs and things like that can seem very overwhelming so just kind of doing things in little steps can seem a lot more manageable*” (K9). |  | Practitioners emphasised that it is important to start with considering basic needs before moving on to beginning to work towards longer-term aspirations and goals:   ”…*And I think what's missing for a lot of NEET young people is just really simple stuff about kind of life hygiene and regulation, that sadly really needs spelling out and that needs to be the initial kind of focus is just saying, you know, life works a lot better if you have some discipline around sleep, some hygiene, sleep hygiene, some like you eat some meals at regular times*” (S19; NHS).  Without the meeting of basic needs, practitioners emphasised that it might be difficult, or young people may not feel it relevant, to work on hope, “”*You can talk to me about hopefulness Doc, but what I need is a place to sleep tonight”…sometimes you can talk about hope and hopefulness and it… might sound a little bit unrealistic to some people”*, (S11; NHS).  Practitioners emphasised that enhancing hope for NEET young women will be a necessarily gradual process, that begins implicitly; “…*it’s a drip-feed, over a long period. Because a lot of our young people…haven’t been handled very nicely by the world, and they don’t always respond well to pep talks shall we say*” (S4; non-NHS). Providers could implicitly build hope from intervention outset through using positive language and conveying their hopes for young people. However, it was advised not to begin with very in-depth conversations about hope, because of its abstract nature and the potential to overwhelm young women at an early stage of the intervention. Practitioners advised that foundational work was needed to build young women’s sense of positive self-regard and resilience before they would be able to engage in work to build their hope and identify their goals:  *“…we do work with young people in terms of looking at their own personal qualities, as their strengths, but also identifying the things in their world that are going well. And it’s about bringing those things into a young person’s conscious awareness…Then we can look at goal setting rather than going straight into the goal-setting when somebody’s on the floor and struggling with negativity. You have to have that hope in place first to provide that bit of motivation and a driving force…guess the trying to perhaps get going on the goal setting too quickly, that can sometimes feel a bit overwhelming, I think”* (K5; non-NHS).  Discussions about its meaning, and the enhancement of hope, therefore needed to begin slowly and within the context of trust having been built in the supporter-supportee relationship:  “…[emphasise] *that we’re not giving up on this and we're with you now, but you wouldn't have such an in-depth conversation I don't think about hope at that point. You have to get some degree of safety in their lives and that's both physical safety and also psychological safety before you could progress to something which is a little bit more difficult to put your finger on*” (S11; NHS). |
|  |  | Behavioural components | Young people spoke of the social benefits of meaningful activities such as volunteering or joining clubs. Being supported to identify these activities helped them to engage and benefit by extending social groups and peer connections. There was a positive affective attitude regarding in-depth discussion with another person regarding goals; “…*it was nice having like, to be able to speak quite in detail about what my goals would be*” (S16). Young women emphasised the helpfulness of being supported to break down goals into manageable steps and reflecting on progress regularly:  “… *letting me explain what worries me and then coming up with small ways just to try and help me reach a final goal of, even if it’s something as simple as like going for a walk, they try to take small steps and every week like ask me how I felt about that and if I can go like a little bit further or just see how I was affected by it*” (S10)  It was additionally considered helpful to have ongoing encouragement to keep progressing through these small steps, including using evidence of past successes to build motivation and self-belief:  “…*just talking through it and like they’d – I get given homework like every week to try and do where I practice like doing small steps and I feel like it was important for me to like have someone to talk to about that …a little bit of a push to try new things to get more comfortable with doing things I don’t like*” (S10)  “*I think there’s a lot to say for setting goals and then, when you achieve them, using them as evidence that I can get over a challenge or I can overcome any barriers....Yeah, I mean it’s quite scary beforehand to think about facing a challenge, but once I’ve overcome it, it is good to be able to say in the future, “This is a hard time, but I remember last time I did it and it was okay.””* (S7).  Young women emphasised that it was helpful if a supporter not only helped them to clarify their goals, but then did some research to ensure they could help identify specific routes to achieve these goals:  “…*to have* [mentor], *who understands the education system, talk me though it and encourage me and be there for me and even though there were things I needed to do myself like certain phone calls…preparing me as much as I can and knowing what was going to happen and be asked was like a major thing*” (S16). | Parents emphasised that the hopeful intervention provider should not only seek to understand and work with a young person to identify their ambitions, but actively support them to achieve their goals; “*It would be basically someone who, together with that person, has identified their strengths, what they want to achieve, what they’re interested in*” (S17).  Parents emphasised that NEET young women often lack both the belief in their ability to reach their goals, and the ability to identify the specific routes to get there:  “*I don’t know whether she’s got the confidence in herself to follow that through. So, it’s never achievable; …how to get for the goal, to that, physically… she needs to be out in the world…because she doesn’t do it. She’ll plan it, yeah, “I’d love to do this, love to do that”,* [but then] *…she shuts herself down so, then you don’t get to the third bit* [of achieving the goal]” (H14).  Thus, support is needed to enhance self-belief and identify goal pathways. It was identified that self-belief can be scaffolded through engagement in enjoyable or meaningful activities, including hobbies and social activities. Building self-belief through these activities was thought to scaffold later EET engagement:  “…*some kind of structured programme, perhaps taking her out to an external gym or some other kind of hobby-based, art class…setting that up and building her confidence and when she’s then, when she’s ready to engage in something a bit more formal, then get her into the education...You know being confident in herself, in her self-identity you know and also you know, achieve things*” (S17). | Practitioners emphasised the importance of supporting young people to identify and support meaningful goals, and to break down these goals into incremental steps and help identify practical means of completing these steps:  “…*our role might be in terms of hopefulness to find them practical solutions to things that are holding them back in their life and let them see that there is hope beyond not knowing where they’re going to be sleeping that night, that they can get back into collage. And at this age they could do the lily pad thing, you know, you want to get to that island in get middle of the pond, it’s too big a jump for the frog, so you’ve got lily pads in the middle, you jump from lily pad to lily pad, and he gets on that island*” (S4; non-NHS).  “*What we'll do…with the goal setting is break down into manageable chunks…because otherwise it can all be very overwhelming*” (S11; NHS).  It was felt that recording achievements, such as through an app or report form, would act as a reminder of ongoing progress, as well as helping to identify, connections between behaviours and outcomes and barriers to achieving aims:  “*You could have some sort of setting-up meeting triage either face-to-face or online like this, and that introduced young people perhaps to an online resource…could they do a quiz? Could you gamify this in some way, so, they say “Right, here are the behaviours of people who kind of get on pretty well. They're not drinking four cans of tenants and smoking weed till three in the morning”. I'm simplifying, but you know stuff about how you live, what you're doing, whether you've got any goals, when you've got friends, whether you get out, whether you exercise, whether you eat, whether you – you know start to map out where are you in all these things? What are the blocks when you say “Oh, I never eat a good meal”, or “I never go out” or, what's blocking you? How do we start that a journey, so you know, and then maybe coming back with someone and saying “Oh* *great you've done this, okay, so how did you feel about it? What does it make you think? Where do we go now next?”*” (S19; NHS).  Moreover, practitioners stated that hopeful intervention providers would need to gently support young people to identify realistic goals that were aspirational, yet grounded in the reality of what could be achievable:  “I *think hopefulness has to be allied with what is available in reality…it’s not like the ether, you know, you have to offer substance to people’s hopefulness, otherwise it’s easy to lose, and easy to lose faith. And when you attach that to a professional role, you can’t give empty promises to people. So, I think hopefulness is very important as a way of, for professionals and people to understand what that means in somebody’s core if you like. You know, that it’s very important to understand that and important to channel that in a positive way and to increase the levels of hopefulness, but you must attach that to something that is realistic and reasonable”* (K3; NHS).  Providers would need to be able to help young women to explore and mitigate barriers to their intended goals, including cognitive, psychological, and practical issues;  “*I guess just showing them that doors can be unlocked and that breaking down their barriers that they already come with. So, it could be things like nervousness around wanting to go to college but* [being] *completely terrified at the prospect of it and helping them with that. Or they want to get into childcare, but they can't understand how they would get there. Or just trying to break down the barriers that childcare, cleaning and hospitality are roles that a woman has to do. Like you could go into construction if you want to, you can be an engineer*” (S2; non-NHS).  It was important too that providers could help young people to explore their identified goals and consider why they mattered, which could additionally help facilitate re-selection if the initial goal proved unobtainable:  “…*being realistic that you may not get the grades…that you think you need to become a doctor, there’s lots of other worthwhile roles people can have and what’s important to you, you know what is it you want to get from that? Is it you know aspirations of others, is it validation is it money, is it community, you know is it friendship, is it occupation, is it approval from a parent, you know what’s the meaning of this for you in your life and so what is it you want to get from it, and then that could maybe mean we can explore it*“ (K13; NHS).  Practitioners emphasised that goal-setting needed to be personalised and an engaging process for young women to do; “…*you need to make things relevant to people and attractive…for them to engage, …to work with people to plan where they want to get to in an optimistic way*” (S19; NHS). Moreover, it was deemed important to emphasise the importance of progress with respect to these small stages themselves, irrespective of reaching the initially identified ‘end-goal’: “*Most people have an ambition to do something. Even if…they feel they can't get to it, there are many stages on the way that they could get off that travellator and find themselves in a place that's quite useful*” (S19; NHS).  Practitioners emphasised the need for supporters to take all opportunities for helping young women to identify positive progress to help build self-belief:  “*I think that, giving them these smart and achievable targets to then check back in with them and acknowledge that - not just skim over it – like really acknowledge that, brilliant you've really done your first action. You've done it, so that's great - let’s put some other stepping-stones in place. It gives them that achievement*” (S2; non-NHS). |
|  |  | Cognitive techniques |  | To increase feelings of hope, an intervention should look to build self-confidence and focus on identify a young woman's strengths; “*Something which invests in them and their self-confidence and their skills, their strengths, and their potential*” (H17). | A hopeful intervention should explore a person’s understanding and meaning of hope as this can differ amongst individuals. Past experiences of hope, circumstances at the time and associated feelings should additionally be reflected upon.  “…*you would have to first of all establish a common ground for a definition of hopefulness because everyone's definition might be different. Like I said, it might be I hope that tomorrow is better than today, or I hope I get to be a NASA scientist. You know, these are two very different… maybe the concept is the same of something being different and better, but individuals might not understand it the same. So, I guess from the start, you would have to have some sort of way of exploring the individual’s idea of what hopefulness is”* (S11; NHS)  *“I think it would be about using…real life…powerful examples of hopefulness and what that means, and what that feels like and you know thinking about when was the last time you had that feeling of hopefulness…* *what was going on around that time? …What's different now? What could be brought back into your life that could help with that?”* (S12; non-NHS).  To gauge current feelings of hope and consider how to increase it, influential factors such as mental health, wellbeing, physical health and substance misuse need to be considered:  *“…it has to incorporate past experience, it has to incorporate present mental state…something around physical health and wellbeing and included in that would be substance use or how well you're looking after yourself, because that again will have a negative impact on your hope*” (S11; NHS).  Techniques for increasing hope could involve using “*motivational interviewing…and just giving solid encouragement, you know, “Come on, you can do this."*” (K3; NHS). Moreover, using techniques and activities involving psychoeducation, strengths-spotting, identifying interests, positive self-talk, and coping strategies enhancement were considered essential to scaffold self-esteem and positive identity, ultimately facilitating hope:  “*So, it's about empowering them. So, it's resilience, isn't it? So, resilience is the belief that you can cope with the negative things that are going on in your life. So, if we can give them the knowledge that they are capable of managing their negative emotions, they are capable of developing coping mechanisms, then that might give them some hope*” (S18; non-NHS)  “*I think more focus on self-esteem and building feelings of competency…* [building] *that internal locus of evaluation as opposed to the external… the seeds can be planted at that age. But I think a lot of it is about letting go of negative self-talk and understanding how that affects you emotionally…introduce things like affirmations and explain…how the brain works basically in terms of things like positive affirmations that they’re not just cheesy things to do, but they do activate certain centres of your brain to start absorbing positive messages into your belief system….it has to come into their conscious awareness first that that is how they believe themselves to be and then look at what forms that belief, what negative messages we send and if you want to change that, you have to be the person who tells yourself the good stuff…looking at what is their strength-based stuff, what is working, how have they coped with what’s happened to them…all those things we try and eek out of a person, so they start to have a more hopeful sense of identity”* (K5; non-NHS)  “*I guess just talking about what their interests are, like just tell me a bit about you. I always find that they're really surprised about that when you do their CV… and that you know they dread it…they’re just like, “I’ve got nothing to add on there, there's nothing about me that's good”. We can obviously work around that and looking at helping them build that and help them word it correctly. But I’d be like, “What are your hobbies and interests?” and they’d be like “Why?” I’m like, “Because it's so good to get a picture of who you are, what you do in your spare time and that is a value” …So, I think that's always really good to focus on when I’m speaking with them too*” (S2; non-NHS). |
|  |  | Interpersonal factors | There was a general encouragement of support for young women to increase their social connectedness; “…*maybe put some emphasis on the kind of like social connection, that kind of thing because that’s been quite helpful for me*” (S6). There was a particular interest in meeting other NEET young women, as this was found to help reduce a sense of isolation and self-stigma:  “…*to be able to meet with other young people who either have dropped out due to similar issues or struggled to attend or had to then be home-schooled and things like that, that was really big for my self-esteem to understand that there were other people in my position*” (S16).  Communication skills was identified as a potentially relevant intervention component; “…*sort of building assertiveness*” (S7). Young women additionally emphasised that there was something beneficial about the involvement of others in working on their hope specifically. The suggested means of doing this included encouragement to share goals and progress with their relatives and friends, the intervention provider working with families to discuss their frustrations and encourage use of hopeful and supportive language and behaviours, helping young women engaging in the same intervention to connect with each other informally, and more formally running group sessions as part of the intervention, for example:  “*Because I definitely find that motivating if I tell someone else that I’m in treatment with, “Oh I’m going to do this,” and then we can support each other to achieve that goal*” (S7)  “*I think for some people…it would have been helpful if someone had been able to speak to my family… there were times when* [my mum] *would naturally get frustrated with me about* [being NEET], *and so I think …her being spoken to from someone else’s point of view about how it affected me and things like that, I think that would have helped. And also, would have probably helped our relationships stay as healthy as possible*” (S16)  “…*when I would meet up with them we would go to a library, two of the people I met from* [the youth service]*, and I would sort of push them out of their comfort zone and also be a motivator for me…it was a helpful thing where I wanted to help them, and they wanted to help me, but we also wanted to meet each other*” (S16)  “*I like the idea of sort of goal setting, working towards that, like holding each other accountable*” (H7) | Parents identified that forming more friendships would be a relevant part of a hopeful intervention; “*I just want her to meet other girls and make friends*” (S14). One parent suggested that engaging in activity with other people would help scaffold identity development; “…*developing that better sense of positive identity through that positive socialisation*” (S17). | Practitioners emphasised that social support scaffolds hope, but that NEET young women be particularly isolated; “…., *they are not in education, not in employment, not in training, so their social interactions are also very limited*” (K5; non-NHS). Thus, a hopeful intervention would be one which prioritised enhancing social support, not just encouraging autonomy and self-belief:  “…*support should help you to be independent, but also interdependent. Because if we just go fully for independence that’s not real life. Most of us are interdependent on other people for support, so most of us need other people in some capacity, whether it’s family friends or professional people, none of us are truly independent unless you’re, you know some off-grid person living in the middle of nowhere. We all need other people. So, we could push too far in independence, but also inter-dependence is important as well”* (K2; non-NHS).  A hopeful intervention should help young women both reflect on how to engage in hope-enhancing relationships specifically, and how to build larger social networks more generally. The first component would involve the exploration of how relationships affect wellbeing generally, “…*so they identify “This is a healthy person in my life”*” (K5; non-NHS), and specifically how they engender hope:  “…*and then also it would have to look at something about those relationships with others… so it's working out the kind of connections and relationships people need and the kind of role models or mentors or peers that they would find who could accompany them on that journey of re-establishing hope and being hopeful*” (S11; NHS).  Practitioners emphasised that as families and peers around young women can exhibit high expressed emotion and encourage young women to stay NEET, “…*they may come from systems, networks that have no hope for them at all, and they’re quite critical of them*” (S3; NHS), then it would be helpful if the intervention could try to somehow augment the system to become more hope-inspiring; “…*if you’re going to be dealing with females, it would be great to try and change the attitude of the people around them, or just for it to be delivered in a more supportive environment*” (S4; non-NHS).  The second component of a hopeful intervention would be supporting young women to build their social networks, engaging in relationships and activities with other people:  “*I think it’s getting healthy social networks, isn’t it?*” (K5; non-NHS)  *“I think what keeps themselves sustained is probably really healthy support networks, and you know they do things like singing groups*” (K13; NHS)  “…*if you can build a little group that then meets up, you know, or WhatsApp’s each other or whatever to say, “Oh, I'm having a really crappy day”, “Oh, sorry to hear it,” you know, a bit of peer support in the group can be a good thing*” (S19; NHS).  The intervention could then help young people with their confidence and communication skills in order to manage social relationships and advocate for themselves with respect to further support-seeking:  “*And also, young people I guess getting a stronger sense of who they are and their boundaries and not being reliant for their self-esteem on other people perhaps*” (K5; non-NHS)  “…*for them to feel that somebody’s there to sort of be there for them and build their skills up around, you know, resilience and believing in themselves and their confidence and communicating. So that actually along the lines that they can communicate their needs or their worries or their barriers so those can be addressed by services would be fantastic*” (S5; non-NHS). |
|  |  | Non-specific factors | It was important for young people to feel supported, understood and not judged or patronised in interactions with an intervention supporter:  “*I mean you want someone who’s quite non-judgmental, and who doesn’t kind of hold an awful lot of inflexible beliefs*” (S13; )  “*… just someone who wouldn’t be like “Oh yeah well, you’ve got to deal with it basically”*” (K8)  “*… don’t talk to me as if I’m a five-year-old. I’m not anymore stupid because I didn’t finish school*” (S16).  The person delivering a hopeful intervention should be able to “…*hold the hope for you when you can’t hold it yourself*” (S7), because being believed in makes young women feel hopeful; “…[to] *have someone believe in your potential to change, that makes you feel so much more hopeful about yourself improving and the situation improving*” (S16). Moreover, providers need to convey enthusiasm for their work; “*People that are actually… so actually enjoy their job, like most of the help out there, now, it just feels like you’re a burden on them, so you don’t want to go to them*” (K7).  The intervention provider should be sensitive to the needs of young people, able to actively listen whereby the young person feels like a “…*regular human being*” (H16). There should be a friendship-like quality to the relationship with the provider working alongside the person receiving the intervention; “…*having an outside voice that you trust and feel like you’re almost like friends with, that helps a lot*” (S16). Providers additionally needed to be able to be responsive and understanding to young people’s varying capacity for engagement; “…*they have to understand that sometimes I might just not talk and that would be okay. I think it’s just understanding”* (S13). | Parents emphasised that gentle encouragement in the context of an ongoing supportive and consistent interpersonal relationship was helpful in facilitating positive change:  “[Employment advisor] *they’d see her every week and, they’d say, “Right how are you doing with this and that,” but then, you know, if she wasn’t kind of doing it, they’d give her a little push you know, in a really nice way that didn’t kind of put her off or anything like that, they would just kind of suggest you do this and do that, you know so they were very helpful*” (K11).  The intervention supporter needed to exhibit authentic empathy and care, with good communication and interpersonal skills considered important facilitators of hope;  “…*they generally talk to you as if you’re just another case. And I don’t think that’s the way to do it. So my daughter is not another case*” (S14)  “…*validate people, don’t just say you care, do care, and communicate it and show it to people*” (S17).  Parents identified interpersonal continuity as very important; “…*it’s not got to be, “Oh we’re closed this week,” or “Somebody else is coming in this week,” that’s just not going to work*” (S14). Parents emphasised too that observably following through on promised actions was considered important;  “[Services] *they pay it a lot of lip service, but they don’t actually enact it. So, it’s not just the absence of the provision of anything positive or following through on what they promised and say they will do…deliver on what you say you’ll deliver, be reliable and be consistent*” (S17).  A more assertive outreach style of engagement was considered needed for this group:  “*She had all these different teams and departments that could help her, but they left it to the young person to contact them if they need help. She was not doing that, or she was forgetting to do that, or she was forgetting to respond, they would take that as the fact she didn’t need help anymore*” (S8). | Practitioners emphasised multiple important non-specific factors they believed important to an effective hopeful intervention. Practitioners argued that the enhancement of hope was predicated on providing genuine empathy, and thus it would be of paramount importance that the provider be attentive and validating:  “…*if you break it down what hopefulness means, it's really attending to a person no matter what their situation is, it's having compassion, …it needs a proper listening, proper attending. And sometimes you can instil hopefulness by not speaking, you're just sitting beside somebody and listening to their story…sharing their plight…I think instilling that hopefulness is about trying to resist being prescriptive. It's about having real empathy*” (K4; non-NHS).  The provider additionally needed to be non-authoritarian and non-judgemental, and not patronising; “*I think young people don't want to be treated like children, they want to be spoken to at their level*” (S1 non-NHS). Moreover, whilst the professional needed to be hopeful and scaffold hope for young women, being overly optimistic was seen to be potentially invalidating;  “…*trying to feel fluffy and hopeful and wonderful and you know “You can do this”, you know they might kick back against that a little bit…some people like to be more practical rather than reflective*” (S12; non-NHS).  Interpersonal continuity, “*Consistency of who their advisor or support worker is, I think that again that's really important*” (S1; non-NHS) and consistent regular support were important; “…*follow-up calls just to make sure everyone is all right, even a courtesy call before the session*” (S18; non-NHS). It was important to have clear expectations at intervention outset.  An assertive outreach style approach was recommended, in the context of patterns of disengagement common to young people with complex difficulties such as NEET young women:  “*I think we need to strengthen the community workers to go and meet them, the clients, that clients, because the clients are not coming to the services, that’s the main worry that we have in all these districts*” (K1; non-NHS)  “*Yeah, they might be okay coming in for a couple of sessions, but then they have a bad day and then they don't want to go, and then they feel like they can't go back because they've they didn't turn up one day…So, it's really important, if that happens, to let them know that it's okay and it's okay to come back, “You haven't messed everything up”, “You're not going to get detention, you're not going to get into trouble”, which is what they're so used to…giving them that information that it's okay to not turn up or to make mistakes, it doesn't mean that the support is over…And then usually when that happens, maybe next time you might get text saying that they can't turn up because they're having a bad day kind of thing”* (S18; non-NHS).  In addition, including in the context of potential disengagement by the young person, it was considered of paramount importance that the intervention supporter follows through on promised actions:  “…*it's writ large that if you say you're going to follow up, you do, you do it on time, you do on the right day, at the right place and if the person isn't there, well then that's one thing but if you say you're going to be there and then you cancel at the last minute, or you forget or don't go, that can be incredibly deflating and create hopelessness and feed into probably a lifetime of experience that that young person has of being let down*” (S11; NHS). |
|  |  | Additional activities | Young women felt that a hopeful intervention should include practical skills and information to support EET activities, everyday living and help-seeking:  “…*more like life skills and skills that could help you in a job*” (K9)  “…*ideally there would be more sort of skill-based, maybe workshops or something to maybe prepare someone for the kinds of things that they would like to do, like whether it’s just CV-writing or sort of more practical things*” (S6).  Interventionists should additionally have knowledge to help with navigating access to EET opportunities and the skills to help identify appropriate activities;  “...*to have like a person in education who understands the system and will actually be able to find things that will work for you, instead of suggesting things when you’re not ready for that*” (S16).  The act of talking about EET activities was itself thought to be hope-inducing; “…*like they were talking about jobs and volunteering, and it made me feel more hopeful as well*” (S10). Similar benefits were identified with respect to hearing about the occupational experiences and achievements of other young women:  “…*at university, I did this programme…where they offered weekly workshops and…they invited quite a few young women to come in and talk about like how they achieved what they had achieved what they have achieved and things like that and overcoming barriers and things. And there was a lot of open discussion as well, so that was really interesting and useful*.” (S7)  Some young women felt that a hopeful intervention should target mental health problems, because these affected a large proportion of NEET young women; “*I guess it would be helpful to look at some skills maybe for managing things with your mental health*” (K9). | Parents expressed the belief that time spent in EET activities conferred hope; “*I think the more she does, the more hope she probably will have, and particularly gaining employment, paid employment*” (K11). Therefore, it was important that hopeful intervention providers were able to support increased EET activity:  “*I think you know some kind of occupational, or job or education based-brokering so matching someone to what could work best for them in moving them forward in education or occupation*…*look for what’s available in the community, either you know training-wise, education-wise, or vocational-wise, and acts to match them up as it were, and support that transition*” (S17). | Practitioners suggested that activities involving the use creative arts might be particularly engaging and well-suited for a hope-focused intervention:  “…*I can imagine lots of the young people we work with actually kind of almost drawing pictures, making it a very creative thing about hopefulness. Some of them are very much – they’ll describe things in colours and things like that, and I can imagine that would be a really, kind of positive discussion to have about being hopeful*” (S9; non-NHS)  Activities such as hosting employer talks, supporting young women to gain work experience or attend work environments would increase aspirations and hope around employment. Help with practical skills such as applications and writing CV was considered helpful as well as information about available resources and pathways:  “...*if it is about NEET young people specifically with the goal to get them into some form of education, employment or training, it could be raising aspirations by you know hosting employer talks or university…not necessary university…but getting them in that place where they can see themselves in in a sector, so they can really get hopeful towards – “Ah actually I can see myself doing this and now I know that this is what it looks like, it's worth the work I need to put into it”. I think everything just seems like a concept, they can't picture themselves in that environment because they might not have anyone else in their family or they've not had work experience. I think, being able to meet employers in a sector or go to events where they can find out more about different sectors...So, I think that might be something that's really good, getting them to be able to see what options there are and do activities and more work experience, but with incentive*” (S1; non-NHS).  Practitioners additionally emphasised that it is helpful if a more multisystemic approach can be used, including, for example advocating for the young person with occupational activity providers:  “…*if the young person’s saying, “I want to go back to school, but I'm struggling with X, Y and zed,” and we can liaise with the school and say, “is there any way around managing that? is there something we can do together to make this work?” So again, tying in the opportunity with the hope but that's not direct*” (S3; NHS). |
|  | HOW | Primarily one-to-one intervention, with opportunities for group components at preference | Individual variation was noted with respect to preferences for the mode of intervention delivery. Advantages and disadvantages for both one-to-one and group modes were expressed. These included that one-on-one interactions could feel more private, and many young women find group interactions very anxiety-provoking; “*I don’t do people anyway, more like just in big groups and meeting people and I just can’t* [cope with] *things like that*” (K8).  However, groups could diffuse the sense of overwhelming attention on the young woman as the intervention ‘receiver’. Therefore, both modes having the potential to feel more intense to different people:  “*I guess if it’s kind of more intense if there’s maybe a lot of people there … to do it with other people that you don’t know and that made me anxious…it’s things that have put me off have been like…doing a lot of workshop things*” (K9).  “*…some people find it easier if they’re in a group because they don’t feel as strained because they’re not just the only person getting concentrated on, but I think some people might appreciate or want that one-to-one help more because they don’t want to share everything with everyone else, they want to be private and they want that one-to-one help, not help with everyone else”* (K7).  The best mode overall, therefore, was deemed to be one in which both one-to-one and group modes were offered at preference; “*So, I think maybe a bit of both, depending on the person’s situation*” (K7). | One parent spoke about how their daughter only responded to one-to-one support; “…*one-to-one, that’s all I can say…or nothing works*” (S14). Another parent advocated for group-based sessions, due to the perceived power of shared experience:  “*I guess like-minded young girls, because they all are…not working, or training or employed or in education…and that I think would give them something to have in common with which would then build their confidence up…it’s a really baby steps but you’ve got to start somewhere*” (S14). | Some practitioners believed that a hopeful intervention should be delivered on an individual, one-to-one mentoring basis; “... *I think in the form of one-to-one mentoring I think would be really good*” (K15; non-NHS). However, groups were considered a powerful means for delivering therapy:  “*I think small groups sort of four, five people where they’re sitting around a table and they’re talking and you’re giving them a structure to help explore themselves and their own barriers*” (S5; Non-NHS).  Groups were considered to be challenging, however, especially for NEET young people, meaning that engagement would either be slow, “… *it does take a while with this type of age group, to build a rapport, especially the ones that are NEET, because they feel nobody cares, to be honest*” (S5; non-NHS), or potentially impossible:  “... *I think you're going to struggle to get people that are already disengaged to come together into one place, because they may be too chaotic…too anxious or…don't have the support networks to engage in such things*” (S3; NHS).  “…*what hasn't worked with NEETs ever is getting a load of NEETs together in one place, it's just not going to happen. Even with copious amounts of pizza or vouchers*” (S2; non-NHS).  In addition, individual needs may be swallowed up in a group format; “*Not in group really because they don’t come up with their need- it should be definitely individually* [delivered]” (K1; non-NHS). Moreover, it was considered potentially unsafe to bring NEET young women, especially from the same geographical area, together in a group; “…*it can cause safeguarding issues… there are family feuds going on…if you're talking about a small age group of 18- to 24-year-olds, nine times out of 10 they'll know each other…it doesn't work for the young people*” (S2; non-NHS).  Thus, practitioners emphasised that a blended approach, always starting with one-to-one delivery and then moving to incorporate some group work at individual preference, would be the best model; “*So that's what I’d say, starting with one-to-ones…and then go on to maybe do some group activities, whether it's visits or sessions*” (S1; non-NHS). |
|  |  | Inclusion of some self-directed components |  |  | Including self-directed activities as part of the intervention was a suitable approach for at least some young people, “…*some people react really well don’t they to just looking at things themselves and just working them out*” (S4; non-NHS), and could make a space for young people to feel empowered:  “*…some of them are capable, and…. I think young people need to be empowered to do things by themselves as well. There's got to be some kind of skills being developed*” (S18; non-NHS).  Moreover, self-directed components could enhance accessibility of the intervention for NEET young women who may feel unable to engage in a supported intervention or to access something within typical office hours:  “…*a digital offer where people work through modules on their own, and I think there's the huge advantage of that for young isolated young people that are able to access things remotely and at the time that you know when they're up at two in the morning feeling the anxiety and frustration, that's when they can get on and get support and I really like that. And you know you work with NEET young people whose routine has turned upside down and they're awake during the night and asleep during the day, those kinds of things. So, it increases the accessibility*” (S3; NHS).  Self-directed aspects could be completed within the context of a self-assembled group of young women to enhance motivation in the absence of formal facilitation; “...*you could almost build something that young people themselves could do as a group, you know, get together in some way and do these things*” (S19; NHS). |
|  | WHERE | Accessible within range of services, as part of existing care or standalone intervention | Young women identified a hopeful intervention as something that could fit in different parts of the youth support system; “*It can be used at any time…anywhere. I think anywhere could do with the intervention*” (K7). It was emphasised that it would be helpful to be signposted to a hopeful intervention from a variety of other services, considering those that NEET young women may be having some form of contact with anyway:  “*… signposting from people who do know about these things. Like I don’t know from other mental health services or GPs or things like that just to make people aware that those services are available*” (H7)  If not directly offered within the services themselves, GP and primary and secondary mental health services were considered to be key referral points able to encourage uptake of a hopeful intervention, for example using printed materials and verbal discussion to stimulate awareness and engagement:  “I *think in terms of attracting them like probably a lot of the time it just helpful to be aware of these things, like sometimes it’s quite difficult to find them online and things, and I think in terms of not wanting to join, I suppose those who might benefit from it most if they are, like already in contact with mental health services or even their GP if they could sort of make these kind of suggestions if they’re aware of it then that’s sort of another way to encourage them*” (S6).  Young people emphasised the importance of interventions being accessible, both with respect to simplicity and speed of access:  “…*it’s very difficult to feel hope when you’re told that you’re at the back of the queue and it’s a year waiting list for treatment…it almost becomes like a self-fulfilling prophecy of “Oh well they don’t care about me, so I don’t care about myself,” type thing, I think it becomes, well it can become a vicious cycle* ” (S7). | Parents emphasised that finding an intervention easy to access is itself hope-inspiring; “*I suppose if it’s easy, easier to access these services then you’ll probably have more hope*” (K11). Parents emphasised too that long waiting lists undermine hope and positive self-regard; “…*as soon as possible, not this year and a half to years waiting list to actually see someone, because I think by that point it’s too late*” (K11). | Practitioners stated that a hopeful intervention could be considered within the remit of and of interest to a variety of different services, as well as being a standalone intervention:  “*But you know maybe an optional thing for organisations like that, aspirational*” (S4; non-NHS)  “*I think there’s lots of organisations, a cross-section of them, that would really want to be involved in helping to deliver that because they, you know, they realise there are so many females that are just sort of left by the wayside really… I think it would be very popular, and yeah that kind of befriending I think there’s lots of volunteers out there who would be willing to help out with delivering it*” (S9; non-NHS).  Practitioners emphasised that young people should be able to access the intervention from multiple points in the system, with as many trained providers as possible:  *“I think the more people that can have access to being trained to provide this intervention, the better …If you had the people with the confidence in this approach in enough places then you're going to be more likely to reach those young people that are really disconnected from services*” (S3; NHS).  It was suggested that a hopeful intervention could be delivered by health and care services, including primary and secondary care NHS, and social services, and local authority youth and employment services:  “…*there's the Employment Service, who I think that would work really well with, working with those young people. I think there's third sector organisations, I think there’s the social care staff that would really benefit from that*.” (S3; NHS).  As an important support service for young people, it was suggested that CAMHS staff should definitely be trained to deliver the intervention: “…*inevitably young people in those situations will come through CAMHS, so having staff in CAMHS that have the knowledge of the intervention to use alongside anything else they're doing*” (S3; NHS). Specifically, it was suggested that a hopeful intervention would fit with the current digital platform being used in CAMHS:  “*I'd also be keen to be looking at any packages, intervention packages you develop, because I think there are places certainly within the services I manage where we could embed those if that was doable. We might be able to put it in …our digital platform, they then have access to that for life*” (S3; NHS).  Additional services suggested included social services, homeless services, police, street triage, ambulance services, and educational organisations; with the emphasis on signposting and referral, as opposed to direct provision:  “…*first contact services, it may be very different. I mean they could be more housing services, mental health services, maybe there could be alcohol and drug services that means they could be having multiple needs, that group may have multiple needs, so they can emerge in any place in the society. Not in one place. So it should be from any service that your service should be open to referrals from any service*” (K1; non-NHS)  “…*universities and things like that, and colleges. I think people who are going to see people in distress are probably the local authority, the surgery, charitable sector and education*” (K2; non- NHS)  “*So, police might be interesting in sharing with them that this intervention is available and where they can direct young people. Street triage at night, those kind of services that will come in, ambulance staff come into contact with these young people...They don’t need to provide it but just to have the knowledge that it’s out there and where young people can go*” (S3; NHS).  It was emphasised that young women should be able to self-refer to the hopeful intervention, in addition to service referral pathways, “…*how it’s referred to could be both self-referral and professionals*” (K2; non- NHS), especially in the context of many NEET young women not being able or willing to engage in any services; “…*there will be some young people that feel frustrated by the fact that they're not employment or education, will* [not] *go to the GPs* [or] *use traditional routes…there will be a lot that much less open and more suspicious and wouldn't go down those kind of routes*” (S3; NHS). |
|  |  | Online presence essential | Young women identified that being able to find out information about an intervention provider online was helpful; “*I think possibly more, well, not more ways to contact them online, but make it more clear where to find these details, like the contact details*” (S6; ). | Parents agreed that intervention options should be promoted on the internet because; “...*young people are always on the internet*” (K11). | Practitioners emphasised that it is essential for interventions to have an online presence, at least for promotional purposes if not for delivery. An option for online delivery was recommended; “…*it would be possible to develop something digital you could do, that could be really interesting, co-producing with people*” (S19; NHS).  “…it's really difficult to navigate, it can be confusing and also you may *not fulfil the entry criteria for X service, Y service or Z service…Which is why I think the work that we should be doing and are doing is around simplifying things… I think with the advent of digital technology as well, increasingly young people can access quickly … that's more 24/7 but also offers a portal into the service, I think that's a good way forward*” (S11; NHS). |
|  |  | In-person sessions should be offered in accessible community setting | Young people foregrounded accessibility as the key consideration for in what setting a hopeful intervention should be offered. Fundamental to the notion of accessibility was ease of travel:  “…*it’s things that have put me off have been like having to travel there*” (K9)  “…*accessible for a lot of people, so maybe somewhere near a train station or a bus, something like that, fairly central*” (S7).  Young people emphasised too that the most suitable spaces are those that feel confidential and welcoming:  “…*it was in a room where you were just with another person, and it was all like confidential and you were just like talking it out. I think that sort of thing was very like welcoming and helpful*" (S10). | Parents emphasised that intervention sessions should take part in easily accessible venues, that did not require extensive travel; “*School or within that local community and easily accessible, I think easily accessible would be really helpful…not too far*” (S17). | Practitioners recommended that the hopeful intervention be offered within “*non-stigmatising, free access”* (S19; NHS), “*easily accessible shop-front type*” (S11; NHS) settings in the community.  Settings that were not obviously educational, health or local authority owned or funded were perceived as best; “…*I think it's good that it's not a local authority or health* [service setting], [NEET young women] *would probably be more likely to engage with a service which is more charitable or whatever*” (K2; non-NHS). This was emphasised as important because mental health services are considered stigmatising and may reflect spaces in which young women have had prior traumatic experiences:  “*Because we are unable to get them engaged with the clinic services. So, you know, they're chaotic, they won't attend, or they can't attend because some of them have what amounts to sort of PTSD symptoms associated with previous engagement with services. Let's say they went to clinic and got sectioned in clinic or something like that, there's no way they're going to go anywhere near a clinic again, so we then have to do that work with them on trying to re-establish the relationship*” (S11; NHS).  Additional benefits of community spaces included that they might co-locate other services and resources useful to NEET young women; *“...maybe in libraries as well…because people can access digital stuff there for free, especially if they're NEET*” (S19; NHS). Outdoor settings were considered a positive option; “*As long as you can try and be confidential, like I say, “Do you want to meet in the park?” or ”Do want to go sit on the beach today and we can work through things?”*” (S2; non-NHS).  Nonetheless, community services alongside NHS services, were perceived as needing to reflect on and improve their accessibility. Good transport links was an important consideration; “…*try and find a venue that has good transport links. So often if you’ve got a venue that doesn't have it, it causes so much stress*” (S12; non-NHS). It was additionally emphasised that services attended by other young people with complex difficulties could be intimidating places for NEET young women to try and attend, especially in the common context of issues with confidence and motivation:  “*You do have a youth organization…like a youth club here…they work very hard; however, they do have sort of a reputation for just being... this is from young people telling me… it's quite an intimidating place to go in terms of it being quite cliquey, not because they are intimidating but it's the same faces, the same young people from quite unstable backgrounds and households, a lot of them with complex needs around substance misuse etc. So, for a NEET young person who is NEET through [a lack of] confidence or motivation… that alone is not a certain place that they want to go to. So, that's sort of a barrier as well*” (S2; non-NHS). |
|  |  | Structured, manualised, and modular | An intervention delivered in small, manageable ‘chunks’ was recommended:  “…*taking things one step at a time, because I know life and the future and jobs and things like that can seem very overwhelming so just kind of doing things in little steps can seem a lot more manageable*” (K9). |  | A structured and manualised intervention approach could help to standardised how providers support enhanced hope; “…*we need to agree - it's almost like we need to agree on the same language, the same frames of reference from the first intervention and sustaining it right the way through*” (K4; non-NHS). Practitioners emphasised the helpfulness of working with young people in small and manageable portions, each of which had a specific focus that explicitly pertained to some aspect of raising hope; “*You could have a module specifically on, I don't know what you’d call, hopes and dreams or whatever you call it*” (S11; NHS). |
|  | WHEN and HOW MUCH | Pacing is regular, but determined by participant preference, and duration is not too brief | Young people emphasised that being able to engage in a hopeful intervention at times that suited them was important:  “*So not like being pressured to come in next week and be like, “When’s the best time for you?” rather than, “We have a slot for this time and that time only.” It would be like in your own time*” (S13)  The pace that the intervention proceeded at needed to take account of young people’s individual needs and preferences in addition; “…*therapy that lets me work at my own pace*” (S16).  Young people additionally expressed a desire for a hopeful intervention that was not so brief that they felt rushed:  “*Maybe* [avoid] *like a time limit. I think that just adds a lot of pressure…anything short term is just… more damaging than it is helpful, so I think that would probably put me off*” (S16). | One parent emphasised that the intervention should feel like a consistent presence in young women’s lives, preferably delivered on a weekly basis:  “*I think it should be something with greater continuity, I think it should be a regular thing, say once weekly. And to you know to also continue, perhaps less often through the school holidays, so it’s something they can rely on*” (S17). | A suggested model was a more intensive beginning to the intervention, with a gradual decrease and then a booster follow-up and gradually decrease:  “…*if it was me and I had a golden wand magic wand, I would have a very small highly intensive but for the people who really needed an injection at that time, and then gradually break it down to less active but not not-active…we don’t want to make people be dependent, but people do a programme of support and then it ends and then they haven’t got any maintenance of that, and if you had a session like three months later just to catch up, “How you doing?” and everything, you’re less likely to regress and then need another bunch of more intensive support. So, I wouldn’t say people should stay open for ever, but…like CBT for instance when they have that, they said it’s just a session on CBT and then no one ever follows it up really and then a year later you need it again*” (K2; non-NHS).  However, practitioners encouraged flexibility with respect to the duration and regularity of a hopeful intervention, emphasising the need to not only provide a short intervention irrespective of young women’s needs:  “*I think the important thing is that the person can come at the start of their journey, and then stay and receive a service from the hopefulness project, until they choose, that they feel they've had the launchpad…that the person agrees a work plan, sees how they can actually subscribe to it and how it will be achieved. And then the plan is delivered, and then the person says metaphorically, well, “I've done it now with your help, thank you so much I'm ready now. I feel I can go on the journey myself”, you know, something along those lines, but having that capacity to enable whoever comes into the project to stay as long as they need*” (K4; non-NHS). |
|  | TAILORING | Led by young person’s goals, needs, and preferences | Young women wanted an approach which could be adapted for each person would cater for differences such as age, experiences, background and interests:  “*I believe everyone needs something different to make them feel hopeful and ambitious…something that’s diverse...and like, can cover a diverse amount of people because … someone older…, someone from a different background to me, might not also find the same thing hopeful than I do*” (K7). | Parents emphasised the importance of the hopeful intervention being adaptable for individual young women; “…*it does need to be very person-centred; it needs to be tailored according to the individual*” (S17).  Parents emphasised that young women would be more inclined to engage with a hopeful intervention if it was shaped around their needs and desires. Rather than directed at achievement, whereby the person is measured against the attainment of a particular goal, it should be person-centred and focus on what they consider important:  “*I think it’s got to be not something that’s another test, not something that they’ve got to do well at, it’s not something that’s going to assess them and produce standards and goals that they have to work for. It’s to help them do and move forward with their lives in the way they want to and achieve the things they want to…* *I think it needs to be adaptive, and a learning process so it learns, you know from, as it goes along, so it’s got to be you know adaptive to people*” (S17). | Practitioners emphasised the need to be person-centred in their approach, making space for young people to lead the focus and approach of the intervention as relevant to their specific needs and goals: *“So, I think it’s more about looking at what their needs are, feeding into what they want to happen and being very led by them, I think would be”* (K5; non-NHS). This included not making assumptions about what young people needed most in the moment:  “*But respecting their own goals, so there is equally the temptation to say, well, we need to get housing sort out and they’re well I don't want to get my housing sorted out, I want some help around my anxiety. So, it's really trying to keep it person-centred and you know what does this young person want, even if it doesn't feel quite in keeping with what they appear to need* “ (S3; NHS).  However, one professional emphasised that some young women might not be able to identify their needs and goals, or might feel that to do so is fruitless, and if so, a more directive approach might be necessary:  “”*What do you want to do today?” I bet you ask any fifteen, sixteen-year-old what they want to do today, how many of them will say, “I don’t know?” And that don’t know could mean, because they really don’t know, or it could mean there’s no point me telling you because they’re not going to get it anyway, or it’s because I’ve never had, somebody said to me once there’s no point telling you because they never get it. There’s no point saying what I want, because it’s never going to happen. So, you know, you have to understand the meaning, so what does that mean when you ask, often it’s better to make suggestions than it is to ask a question, “Do you want to try this?”*” (K13; NHS). |
|  |  | Primarily in-person, with remote delivery at personal preference | Young women described the preference for in-person versus online intervention delivery as individual and dynamic. A preference for online sessions was predicated on knowledge and access to appropriate technology; something that was not always considered by providers;  “*I did it the other day on the Tuesday, but they were trying, just trying to get me to go on the Google Classroom thing or something at the same time and I couldn’t do that, my phone wouldn’t let me do that when I was on the, what do you call it, you know the Facetime call thing*” (K8).  Therefore, it was suggested that the hopeful intervention should be offered to young women via in-person, telephone and/or online sessions at their preference; “…*having options to either go there or do it online could be like less intimidating*” (K9). The intervention provider should be mindful that this preference may change on a session-by-session basis:  “...*having options so that if someone’s having a particularly bad day then they can go on like a virtual platform rather than having to leave the house*” (H7)  “…*I know sometimes when I’m having a bad day, I don’t want to be around anyone else but I will, if you ring me, I’ll talk to you for a little bit, like until I’ve had enough*” (K7). | Parents emphasised that in general, young women are very au fait with technology, “…*they’re used to the online, they’re used to chatting to their friends online and all things like that*” (S8), but that some young women may struggle to access online based support:  “*It’s quite a big, with [daughter] I mean a couple of times it didn’t work, electronically, the computer wasn’t working the second week I think for [daughter], and she did, even though she finds it harder on the telephone, you just would hear her ask to repeat what’s they were saying if she didn’t understand, but she did it all over the phone and things and stuff. I don’t think over the phone is as good, but if it’s all that’s available to that child at that time, then actually I think it’s better than nothing. I think it’s just the knowing that someone’s there really, isn’t it?”* (S8)  Parents recommended that young women should have the option as to attending in-person or online intervention sessions; “*I would say both if, basically to have the option to do both would be quite good*” (S8). Parents emphasised that providers should be able to flexibly shift between online or in-person delivery based on current needs; “*To be able to operate in both avenues, or to be able to switch between the two depending on how they’re getting on*” (S8). | Practitioners appeared to recommend, overall, intervention sessions delivered in-person, suggesting that this provides more “*authenticity*” (S11; NHS), and better meets young people’s needs:  “*So, I've recently done a consultation with young people around how they access health care advice and information, and a lot of, the majority of them said that they want face to face appointments, they want that face-to-face thing*” (S18; non-NHS)  “…*young people live and die by their smartphones these days and Facetime etc, but they're still saying you know if I’m going to engage with somebody and talk to somebody, I want to do it face to face first time, if not every time*” (S3; NHS).  Nonetheless, online sessions were considered to be a good means of engaging young people who exhibited patterns of disengagement:  “*We moved to virtual, and we found that actually virtual was working quite well because we would see a lot of non-engagement for appointments and no-shows, but actually when they're in the comfort of their own home that improves. So, I do think continuing with an online platform with this group definitely is important*” (S1; non-NHS).  Practitioners recognised that access to technology is an important barrier that undermines the suitability of online sessions for all young women; “…*not everyone has access to digital and that's one way. So, then you might have to have some sort of paper resources*” (S19; NHS). This was suggested to a particular risk for NEET young women; “…*you might have a problem with NEETs is that they don't have access to a computer…*[or] *might be in a family of five and have one computer*” (S2; non-NHS).  Overall, therefore, practitioners recommended a blended delivery approach:  “…*face-to-face really that is the best intervention, but you know over COVID we have seen that it has been really useful to make our services more accessible, and that’s going to be the issue with people who are NEET for anxiety reasons, than expecting them to turn up face-to-face. Or you know young people might have an anxiety about appearing on Zoom and an aversion to telephones, so the more options the better really*” (K5; non-NHS)  “*I think interpersonal relationships are really powerful in that, so I think having a package, I think a digital offer, I think, ought to be part of any offer that we’re thinking to develop for young people moving forward. I think probably a blended offer and I think different people - if you could have it to the extent that you know, some people could do it fully online and other people could err towards more face to face and then there's an option in between the two*” (S3; NHS).  It was recommended that an intervention should aim to begin with at least one in-person session to build initial rapport and then with the potential to move following sessions online; “…*you could do the first one in person, and the other two online you know that kind of thing*” (S3; non-NHS). |
|  |  | Flexible content and activities, options for active engagement | Young women suggested that in-built flexibility and choice in how to complete the activities of an intervention scaffolds engagement; “*There’s you know options for people to work in a way that suits them better, that definitely helps a lot for me, I can’t really think of anything else*” (K9). | Parents stated that intervention providers needed to understand that NEET young women are not a homogenous group, and how the intervention is completed should reflect this; “…*young people are individuals and what might work for one person might not work for the next person*” (K11). | In the context of the abstract-seeming nature of hope, and young women’s individual differences, practitioners emphasised that providers should be “*creative*” (K13; NHS) at adapting how they delivered sessions to individual NEET young women to fit with their individual needs; “…*you know you have to be flexible; you have to be responsive*” (S11; ). It was emphasised that hopeful intervention supporters would need to be equipped to use different means of discussing the concept of hope and its relevance:  “…*if you talk to someone who is maybe neurodiverse, who is going to be quite concrete about things, that might be a very different conversation to someone who might have more emotional literacy and understanding about their own emotions and the emotions of others, you know, then you could have a chat about hope and hopefulness from that kind of positive psychology point of view, maybe throw in a bit of Buddhist approach to life*” (S11; NHS).  Young women were identified as having different interests, which could incorporated into intervention activities in order to make them engaging:  “*So maybe you do your work and alongside other things that are interesting to people, a few things together little bit. So, you know for instance you could have people who are interested in novels or writing, you know they might want to you know push the bit about hopefulness well novels to help you to feel hopeful and you know, well you know all that sort of stuff so they’re more likely to engage*” (K2; non-NHS).  Practitioners emphasised that activities needed to only invite young women to do things that were achievable, considering their specific problems and experiences:  “…*you need to kind of really give them something to do that’s going to be just that kind of a stretch in their comfort zone, so they have a sense of achievement, so that’s why it’s kind of really important to think about who your cohort is and what you’re going to do with them*” (K13; NHS).  Practitioners emphasised that sometimes, and for some young people in particular, using an option to take the intervention outside was really valuable. Doing so could provide benefits of involving a young person in something new, exciting their senses, and either making it easier for them to talk or offering a non-verbal means of engaging in the intervention activities:  “*You know you obviously have to be quite careful not to bombard them with toxic positivity but to work with that resistance a little bit and find out where they’re coming from. But sometimes it’s good I think to sometimes get out of the therapy room and we do a lot of like focusing on like getting out in nature and doing activities, introducing a young person to something new, it sort of wakes the brain up a little bit, even if it’s just taking a walk somewhere that they’ve never been before*” (K5; non-NHS)  “…*maybe thinking about outside the four walls of the clinic, I think about experiential sensory experiences rather than verbal, I think a lot of young people often have difficulties with talking treatments. And they may not be very, there’s big gaps in their learning and in their experiences and in their development which means that, like I said, asking them to think about a goal might be quite difficult when they don’t know what it is that they want to do or they could do, what is available to them. So, I think in terms of just inciting curiosity, to think about what is around them and what do they like in different contexts, and I think there’s real value in group activities, that aren’t kind of maybe therapy but they are therapeutic*” (K13; NHS). |
| 2: Who are possible non-specialists that could credibly deliver the intervention? | WHO PROVIDES | Supporter is experienced, but not too professional | Having practitioners involved in delivery was seen to offer credence to a hopeful intervention, through ensuring that the person supporting delivery is experienced and assured:  “*I think having a professional there would help with that like hopefulness because having someone who is experienced and knows what they’re talking about, delivering it…it’s easier to believe when someone’s sort of speaking with conviction and experience*” (S7).  However, it was emphasised that a supporter’s ability to form positive ongoing relationships with young women is more important than any professional qualification or experience:  “…*she’s not a therapist and it’s obviously a different type of support, it’s building a relationship with someone who will push you out of your comfort zone but still will let you go somewhat at your own pace and support you in the long run*” (S16).  Some degree of personal and/or professional experience in health and mental health was seen to be particularly important; “*Maybe like people who like have studied like medical, medical professions or like psychology*” (S10). It was seen to be helpful for providers to understand the wider support systems around NEET young women; “*So have someone who has gotten to know you and knows the system*” (S16). | Parents emphasised the important of practitioners being involved in intervention delivery, “…*they’d need to be some kind of professionalism to know what, you know how to help the young people*” (K11), with respect to ensuring intervention effectiveness. Example practitioners included those trained in occupational therapy and experienced in working with trauma, for this could be helpful in “…*improving people’s self-confidence and their ability to communicate and present themselves*” (S17). However, there was additional interest in the intervention being supported by someone who acted as a “*mentor*” (S17). | Practitioners suggested that intervention delivery could be supported by workers who were not ‘too professional’, for example, youth workers, employability workers, and volunteers. Practitioners stated that one key priority for NEET young women to feel heard and that a professional supporter is not necessary for this:  “*I think the main thing for this cohort is that they feel listened to and heard and I don't think that always has to be done by mental health specialists…I think young people don't want to be treated like children, they want to be spoken to at their level and not patronised… so I think as long as the professionals have that characteristic*” (S1; non-NHS).  Authenticity is another key priority, and again is not predicated on professional expertise:  “…*doesn’t have to be an expert. I think the key is making sure that whatever it is, it is delivered authentically. The young people certainly that we work with are very good at spotting a fake…and if you have a conversation about something like hope and it's clear …you’re thinking of something completely different, then young people spot that.* [Whoever delivers the intervention needs to be] *able to do it in a way that is youth-friendly, that is authentic, that they're able to give of themselves*” (S11; NHS).  Moreover, young women may actually more able to have open conversations with someone who is ostensibly not a professional; “…*they should not be a professional ideally because they wouldn’t come and tell their problems then, they won’t come up, they won’t open up*” (K1; non-NHS). In addition, provision by non-expert workers was deemed necessary to ensure a low-cost and scalable intervention with wide reach:  “*It fits well into that rapid or at least swift response*” (S11; NHS)  “…*it’s probably support worker-level, possibly a little bit higher than that or it wouldn’t be low-cost, it would be expensive*” (K3; NHS).  “…*if it’s going to be delivered by no health care workers or non-specialists, it’s going to be at the community level. You could at least access more than 50%, yeah 50-75%*” (K1; non-NHS).  The design of the intervention was important, nonetheless, with respect to being suitable for delivery by non-experts;  “*I think if the intervention is designed right, it should be that anyone could deliver or access it pretty easily, I think it should be in plain, non-therapeutic feeling, straightforward goal setting, targets*” (S19; NHS). |
|  |  | Support occurs in a mentoring type relationship |  |  | Practitioners identified a mentoring type relationship as an appropriate vehicle for intervention delivery:  “*I think in the form of one-to-one mentoring I think would be really good*” (K5; non-NHS)  “*I suppose I was almost imagining …kind of mentors, people that might – you know there’s lots of people that do volunteering in our area, and I think, we have befriending services and almost, if you had something like that, I think that would be very popular*” (S9; non-NHS).  It was suggested that the optimal model could be to create a way of training anyone that the young person themselves felt they best connected with to support the delivery of the intervention, within a mentoring type of relationship:  “*Often, there are a lack of role models for young people outside of health and social care… it's almost better if it was maybe from peers or someone who is in a community role – not in a kind of healthcare provision role - who can then provide that role model for them… mentors, so people who are, they're not considered peers of the young person, but they'll be older people who have been through some of the similar difficulties or have a lot of knowledge about it…Before I trained in psychiatry, I did spend a bit of time locum-ing, and one of the places I did a locum in…all the staff were trained to a basic level in being able to talk to the individual…or the family so that, if they then decided that it was the cook in the kitchen that they connected with, the cook in the kitchen has got some basic training and also has regular group supervision with their peers they work with because you just don't know who someone's going to connect with and I really liked that idea. Similarly, if hope and hopefulness was promoted within a service or a team and everyone was given some basic training, including the receptionist and the cleaner, I think that would be quite powerful”* (S11; NHS).  This type of approach additionally increased the chance that this relationship could persist over time, providing the young person with a sense of relational and support continuity, and make use of all the people existing in communities inhabited by NEET young women that were emphasised as wanting to be able to help them thrive:  “…*they like to know there’s somebody there, the best kind of mentors in the community that I know, you know they come back around again, I’ve had some young people they’ve worked with four or five years ago, and they’re still doing it*” (K13; NHS).  “…*the supported living providers, …the foster carers that have so much passion*” (K4; non-NHS). |
|  |  | Multi-perspective involvement, including near/peer expertise, but with emphasis on interpersonal qualities | It was felt that a range of practitioners and non-professionals would offer diverse insights to the intervention and its delivery. Non-professional peers, or near peers, were seen as able to facilitate a sense of shared experience and modelling of overcoming adversity; “*I think hearing from other people that have been though similar things and have got through it is really helpful*” (S7). However, empathy from those supporting the intervention was more important than near/peer status:  “*I think that it would be nice to be supported from, with someone who’s been through something similar. But at the same time, it’s almost they can empathise and try and put themselves in your shoes instead of just looking at it from a purely factual point of view. I don’t think you even need to have that, necessarily, I think it just depends on the type of person you are and if you have been understanding you have of how difficult it is...so I think having someone who’s been through what you’ve been through can be helpful, and it would be nice if there was another person that could really truly relate, but I don’t think that that’s the only option*” (S16). | One parent emphasised that “…*a combination of both*” peers and practitioners should be involved in delivering a hopeful intervention (S17). Another parent emphasised that good communication and partnership-working is essential. This pertains to both different hopeful intervention providers and other services which could become involved in the young women’s care:  “*I would say the only thing is possibly to work together a bit more…because they’re all in different areas, all in different teams…sometimes being able to tie that together might be a good way to everybody you know, I guess some form of like a of a care plan type thing almost that goes with, that belongs to that person*” (S8) | Practitioners emphasised the benefits of different types of supporters, including different professions and peers or near-peers, being involved in intervention delivery and support:  ”…*a group of different professionals…bringing all their different experience, knowledge and strength. Employability, yes, youth workers, yes, …peer*” (S1; non-NHS)  “*Trainers should be psychologist and psychiatrist as well as some positive thinkers and some employment advisers. And if you can enrol some of the entrepreneurs in the area where you could provide some occupation, employment some kind of qualification and some university or technical, or collage stuff. So that would be ideal*” (K1; non-NHS).  Additional benefits of involving peers specifically included that practitioners felt young women would value the peer perspective and find it more powerful:  “...*sharing your own* [experience]*, I think it's so powerful for young people, for anyone. You know someone that's been through that and been there is so much more powerful than you know professionals or someone trying to say, this is how it should be done. I think you know lived experiences is really valuable and appreciated by people*” (S12; NHS)  “…*we also recognize that at times, when people are really down, they will have lost hope. So, we can see that and we recognize that but [say] “We've got examples, we've got living examples we can introduce you to” - I wouldn't say like that but you know what I mean. We can connect you with people who've been in a similar situation who would be able to tell you a bit about how they overcame their difficulties in that they kind of became much more hopeful about the future and achieved things*” (S11; NHS).  Other benefits included that peers or near-peers could enhance hope through modelling that young women’s circumstances can improve:  “…*if they were to be able to see other people from similar backgrounds who have experienced similar things, if they're able to see it done, you know, throughout that thriving, achieving thing, then I think that would give them some hope for their future*” (S18; non-NHS).  Previous intervention recipients were a suggested group to deliver to other NEET young women. Peer-delivery was thought to enhance both engagement of young people and skills of practitioners involved:  “*I’m really keen on getting young people on board because that then attracts other young people…that’s a huge selling point, it attracts others and really good learning for the other professionals, I think. That’s really great for feedback*” (S9; non-NHS).  If intervention providers were practitioners, it was argued they should be as near in age to young women accessing the intervention as possible; “…*youth workers that are not that old …someone that they can that they can relate to, so it’s not some grey guy in a grey suit*” (S5; non-NHS). |
|  |  | Some form of mental health expertise or input | Young women identified that mental health expertise would be useful as part of a hopeful intervention. Practitioners might include counsellors and psychotherapists:  “…*different mental health professionals like not just counsellors but psychotherapists, and stuff like that could maybe get involved …they have different aspects and different insights to different stuff so like, something that psychotherapist said might be different to what a counsellor says*” (K7). |  | Irrespective of the nature of the provider, it was suggested that they should have some relationship with mental health services, “…*a good link to mental health professionals*” (S1; non-NHS), with the ability to identify when more specialist mental health support might be needed; “…*your hopefulness worker could identify that the client need to be referred, that’s great*” (K1; non-NHS). |
|  |  | Non-intensive training of a small number of specific elements | Young women identified that intervention supports should have some training in mental health literacy and non-specific therapeutic skills:  “…*aware of different mental illnesses and disorders and knowing how to deal with them….just listening to them and understanding and trying to help them in the best way*” (K9). |  | It was felt that the person delivering the intervention did not need to be highly trained or qualified in complex mental health issues. Instead, providers would need to understand the importance of a hopeful intervention and how to form positive relationships with and support young people to be able to engage in it, especially in the context of patterns of avoidance:  “…*although people won’t be qualified as such, I think they need a level of training and understanding, empathy, support, mentoring and that, the people that do it. I think the people that are doing the support could probably be, some of them have lived experience.*” (K2; non-NHS)  “*So, training in the actual intervention, but I suppose training in the “What ifs”…when young people say, “Well that's all right, but I can't do that because I'm anxious or because my family” - well, you know, whatever reason, so that's where direct training comes in really handy, I think, because the package - I don't know how simple the package would be if it was a teaching session then I think it could be open to anybody, I guess - but it's sort of an intervention, as such, I think that's more complicated*” (S3; NHS).  In addition, having someone not trained to deliver a range of therapeutic models was considered preferable as they would be more flexible regarding novel ways of working:  “…*you don’t want someone who’s got lots and lots and lots of kind of different models…you want them to have a new way of thinking, a new way of working, they’re more receptive to a new training if they haven’t already got like twenty other model stashed in their belt*” (K13; NHS). |
|  |  | Access to supervision |  |  | Practitioners emphasised that intervention providers should have support and supervision from other practitioners. Supervision was needed to help with the technical elements of delivering the intervention:  “*They would need supervision, they would need some group supervision to support, you know they would need management, support, you know, in terms of you know, making sure that resources and everything's in place*” (S12; non-NHS)  “…*so if it was if it was a young person I was working with and I didn't really know of a service I would bring that into my reflective space with the team ask for their ideas around what services would be you know appropriate if there are any, you know pathways in place that this kind of issue, whatever the issue was, whatever the goal was*” (S12; non-NHS).  Emotional support was additionally needed to help manage reactions to hearing about trauma and negative experiences of NEET young women:  “…*the service providers…they need some time-to-time psychological support also because I know that they are going to listen there listen to some bad experience, negative experience…the service providers also need to have some kind of support*” (K1; non-NHS)  “…*because I suppose a lot like social workers, and counsellors, therapists they kind of got to have that release as well, they’re coming up with, I’ve heard lots of harrowing stories…I think they’ll need some kind of outlet to support them to be very positive, because it’s hard being positive all the time, isn’t it?*” (S9; non-NHS)  Emotional support was considered particularly important due to the potential contagion of hopelessness:  “…*the people who are delivering it having, them having good supervision and support, because it’s exhausting to inspire hopefulness in hopeless people. You need a robust support for the people who are delivering the intervention. You need to think about, you know you cannot pour from an empty cup. So, in terms of, because you know that’s often what happens isn’t it is people get burn-out and they leave and you have a high turnover of staff and therefore, young people never build that relationship …Build your staff skills up, their resilience, their sense of you know, ‘We can do this’, because you don’t want to go, young people to go into a group where the staff are like “Well I’ve been doing this for, nothing works it’s a waste of time”*.” (K13; NHS).  It was additionally considered helpful if the supervisor(s) had some expertise in mental health problems:  “…*could it be that they have someone who's a supervisor that's the mental health professional who can feed into them what next steps or how to approach things? I think that would be good*” (S1; non-NHS). |
| 3: What are the most meaningful short- and long-term outcomes related to hope and its theorised effects on mental health and social functioning, and how can these be measured? | | Nature of hope as goal-oriented, future-focused, with motivational and goal-route components, and an implied sense of actively improving and becoming more positive | Young women described hope as goal-directed and future-focused with cognitive and affective components:  “*Having an idea of what you would like from your life*” (S6)  “…*it helps me set goals for the future*” (S16)  “…*positive thoughts all together and not the negative doubting ones like the ‘I can do it’ rather than ‘No I can’t’, kind of thing*” (K7)  “*…feeling positively about the future, and kind of wanting to experience in life and just feeling like there’s more for you in the future and being excited for it*” (K9).  Hope was experienced as a motivational force by young women, manifesting in increased energy, positive self-talk, and optimistic thoughts about the future:  “*I think I’d probably feel more motivated and sort of more energised*” (S6)  *“…‘“I can do it” rather than “No I can’t’” …that there’s hope...I’m going to get better, yeah things are going to get better and things are going to get easier*” (K7). | Parents described young women’s hope as goal-oriented and future-focused; “…*goals, future, you know...You, you have plans and things you want to do*” (S14). Parents identified both cognitive and affective components as relevant to hope and its effects;  “*They feel a greater sense of motivation or move forward, a greater sense of self belief and personal safety…and confidence*” (S17)  “*Well* [hopeful people] *feel good about themselves*” (S14).  Parents emphasised the importance of hope in young women’s mental health and social recovery:  “*And I think having hope is a massive part of recovery almost. You know because you can see something for your future, you can see you know, okay you’ve had this issue for years, but now you’re looking forward, you know with moving on. Which is what she’s doing*” (K11).  This included an inherent link between hope and positive functioning; “…*you look after yourself, which she didn’t. You, you have plans and things you want to do*” (S14). | Practitioners described hope as imbued with a sense of dynamism; “*I guess there’s a sense of energy about it, there’s a sense of excitement*” (K13; NHS). They identified hope as goal-oriented, future-focused, and comprising cognitive and affective components:  “…*it's very much about building blocks and stages to get to doing something that you want to do*” (S19; NHS)  “…*looking forward*” (K13; NHS).  “…*a constellation of quite similar feelings and attributes, yeah self-belief and so on*" (K3; NHS)  Thus, hope for NEET young women scaffolds belief that they can reach their goals, even if they cannot pursue them in exactly the way that they would want to in the moment; “*There is hope, you know that they can they can achieve…even if they're not in the right space now…they feel that the doors are open and they can get the support and opportunity to really try and reach their goals*” (S12; non-NHS). |
|  | | Hope leads to increased EET activities | By affecting motivation, young women identified that hope then additionally influenced the desire to work:  “*It* [education] *impacts it* [hope] *greatly because if I wasn’t doing either, well if I hadn’t done the GCSEs and if I wasn’t planning on doing the access course, that’s when I feel most helpless and hopeless because there’s no progress at all being made and I would be still stuck in the same point that I had been for years and when you’re in that point it’s really hard to recognise that it is possible to get out of that because of the little support and everything, so it’s hard to focus on the things that you want to do and the fact that things can change, when things haven’t changed for you in years*” (S16). |  | Practitioners identified hope as facilitative of EET activity engagement; “…*hopefulness is good…that grounding is especially important for education, training and employment*” (K2; non-NHS). |
|  | | Hope leads to improved social functioning | Young people described hope as inherently linked to positive functioning and activity:  “*Like I’m fighting to do it, but I want to do it and I want to be able to feel like I actually want to get up this morning, and I actually want to go and do stuff, not being made to feel like I’m being made to do it. I want to be able to feel like I’m doing it off my own back*” (K7)  “…*just feel generally day-to-day more able to go about doing things*” (S6).  Hope was additionally described as facilitating social connectedness; “*And probably a bit easier to sort of to connect with others*” (H6). |  | Practitioners emphasised the links between improved hope and social connectedness, identifying this as a potentially bidirectional relationship; “*Maybe potentially their social networks have improved and all those things kind of play into each other, don’t they? To make you feel more hopeful as a person*” (K5; non-NHS). One professional emphasised that hope manifests as engagement with support in the form of attending appointments and completing homework, including progressing in activities identified as relevant to pursuing personally meaningful goals:  “…*commitment to appointments, you know not falling off the radar. Doing their own… you know we set action plans, so tasks for them to go away and do in between appointments and seeing that they're doing them and not just ignoring them - it's that commitment to their progression*” (S1; non-NHS).  Another professional emphasised how a hopeful intervention could facilitate small changes in daily functioning as part of scaffolding wider changes in mental health and behaviour:  “….*it's sort of trying to remove this sort of Everest approach to life where, okay, you're not going to go and climb a mountain tomorrow. But you might get up in the morning and have breakfast and that's a really good start*.” (S19; NHS). |
|  | | Hope leads to improved mental health/ reduced mental health symptoms | Young women emphasised that being hopeful supports better mental health; “…*my mental health improves a lot when I feel more hopeful*” (S16). Specifically, young women identified that hope helps them to cope with negative thoughts and emotions experienced in the context of mental health problems;  “…*it helps me deal with negative emotions better, because if I just get through this then there might be change in the future and I might be able to do all the things that I want to do*” (S16).  Hope makes it easier to manage mental health problems, reducing the sense of “struggles” (S10) and making recovery feel more possible; “…*if I’m feeling sort of less hopeful, I feel more sort of ingrained in the mental illness and sort of feel that I have to turn to that because I lack any sense of direction*” (S6). | Parents described hope as counteracting the hopelessness that is inherent to mental health problems such as depression, “…*you’re kind of in a dark kind of tunnel …hopefulness to me means there is a light somewhere*” (K11).  Parents identified hope as protecting against suicidality:  “… *from the very, very first time anyone’s ever spoken to [daughter], the doctors, anybody, nothing has ever been about [daughter] not wanting to live, not wanting to be around or anything like that. She’s always so, so from that point of view, I think she’s always, she’s always been a particularly hopeful kid*” (S8).  Moreover, parents emphasised that hope scaffolds belief in the possibility of mental health recovery:  “…*hopefulness means belief that things are going to get better*” (P17)  “…*having hope is a massive part of recovery…because you can see something for your future…you’re looking forward, you know with moving on*” (K11).  However, one parent emphasised that change in mental health problems may actually come before change in hope;  “*I don’t know what comes first, I presume it’s her mental health, you know, recovering from that, depression, anxiety*” (K11). | Practitioners emphasised that hope (or a lack thereof) influenced mental health symptoms, and associated problems such as suicidality; “…*because people who don't have any hope are at increased risk of deciding that well “I may as well end it all”*” (S11; NHS). Hope was considered to promote mental health and reflect better emotional wellbeing:  “…*it’s what creates good mental health*” (K2; non-NHS)  “…*it’s one of the questions we ask in our wellbeing questionnaires, at the start of services and at the end of services, is about feeling optimistic about the future, which I think kind of summarises hopefulness really doesn’t it, in a sense. And you know, yes, to see that it’s a real indicator that someone’s emotional wellbeing has improved*” (K5; non-NHS). |

*Note: S = Sussex, K = Kent.*
