# Supplementary material for: Developing a hope-focused intervention to prevent mental health problems and improve social outcomes for young women who are not in education, employment, or training (NEET): A qualitative co-design study in deprived coastal communities in South-East England
Source: PLoS One. 2024 May 31;19(5):e0304470. doi: 10.1371/journal.pone.0304470 (PMC11142577; doi:10.1371/journal.pone.0304470)
Supplement: S2 Table — (DOCX) [file pone.0304470.s002.docx]

**S2 Table. Data analysis of Phase 2 co-design sessions conducted with NEET young women.**

| **Research Question** | **TiDiER item** | **Additional sub-categories** | | **Key points** | | **Illustrative quotes (labelled with workshop number)** |
| --- | --- | --- | --- | --- | --- | --- |
| i: What format and setting(s) are viable for delivering a brief, low-cost, hope-focused intervention to NEET young women aged 16-24 years in deprived coastal communities? | WHY | Rationale for psychological intervention focused on enhancing hope | | - Primary focus on enhancing dispositional hope, i.e. learning the skills of hope and goal pursuit - No explicit and specific reference made to EET activities – although individuals may identify these as personally meaningful goals, and enhanced hope would likely result in increased EET activities | | “…*it's giving something positive to the person…when you focus on what you want in the future, even if you can't think of it yourself, to have someone with you to help you list all things you can do, it kind of opens up things that you didn't think that you could do*" (W6)  “*I think it would be helpful to understand the skillset I need to like actually achieve hopefulness…making someone feel comfortable and confident enough to think about things they want in the future, then giving them those skillsets, is kind of more important than necessarily making it exactly about like work and careers*” (W1)  “…*rather than the focus being on specifically employment and education, just creating that hope and confidence so that that comes anyway*” (W3) |
|  | WHAT |  | |  | |  |
|  | Materials | Participant workbook | | - Available as paper and online versions - Different informational resources (e.g. videos, lived experience stories, discussions with supporter) for use at participant preference - Accessible for people with neurodiversity - Non-patronising, non-clinical and non-childish language and branding - Use words such as “hope”, “goals”, “manageable”, “achievable, “fulfilment”, “happiness” and avoid words such as “success”, “achievement”, “responsibility” | | "…*it could be quite helpful, particularly examples and stories from people with lived experience*…[a video explaining concepts, e.g. hope] *is the kind of something that could be done towards the start of the sessions…it’s quite a good kind of thing to open with*" (W3)  “*Just because someone who isn’t in education or anything like that, they do know what those like words mean, you don’t need to like make it bright and colourful and like very childish*” (W1)  “*Just like language that is quite good, like hope and goals, because it's encouraging*” (W6)  “…*things like “decisions”, or “responsibility”, something that will probably put quite a bit of pressure or I don’t know something like “success” as well, they’re all quite enforced rather than coming from a person themselves*” (W3) |
|  | Procedures | Accessible and acceptable to young women of diverse backgrounds | | - Acceptable to young women of different backgrounds, including marginalised groups - Accessible at times of life transition - Promote where young people are, including via social media | | “*I think there should be more help, especially like if people have left school and haven’t moved on…then they still can sort of hope or feel like they could sort of get somewhere*" (W4)  “*Maybe like on the Internet, like social media…Maybe at like some local places like libraries or something where they can come like hand out things to people*” (W6) |
|  |  | Gentle introduction to hope | | - Gentle introduction to hope | | “*I like the bit of not focusing on hope in the first, I think that’s good…but I do think the first one being more like, getting to know you and a little bit less of the intervention itself, a bit more relaxed, that that is important, not like throwing someone into the deep end*” (W1) |
|  |  | Behavioural components | | - Identify and increase meaningful activities, focusing on both known and novel activities - Identify and plan how to overcome barriers to increasing activity, including anxiety and fixed routines - Identify and work toward meaningful shorter-term goals - Goal visualisation to help identify goals - Teach skills of breaking down goals into small steps, in a personally manageable way - Review ongoing progress towards identified goals - Help to identify and plan around goal barriers - Helping identify connections between goals - Participants ranked the identification of interests and meaningful activities, and learning how to breakdown goals into smaller components and identify and overcome potential barriers as very important | | “…*the more you do something, or you try different things like activities, it makes you more comfortable with wanting to achieve your goal, like going back to school or being employed*” (W6)  “…*there should be more help there…try to think of different solutions and ways that* [people with anxiety] *either like go there or like things like that*” (W4)  “*Perhaps in terms of behaviour, something along the lines of flexibility or maybe changing routines. Just sort of try and help people stop, like not get into a bit of rut, if that makes sense*” (W3)  “…*they could kind of point ideas as to what people could do, or perhaps ways to access certain things that they might have not heard of before… open up ways for people to either engage in new activities or more social activities, like how to go about it, where they might be able to find stuff like that*” (W1)  “*I think that* [goal visualisation is] *probably a fairly helpful way to do it, so I guess maybe a more calm environment where, because I think if they suggest like a lot of goals in one go, that could feel quite overwhelming, so I guess this is a safer way to do it”* (W3)  "*There was that one* [suggested activity] *about looking at the individual steps like to achieve a bigger goal, saying that that would be like really, really stressful to me, trying to think about every single thing I have to do to achieve one thing*." (W1)  “…*allocate times to maybe take some of those small steps and maybe reflecting on how you’re feeling about doing it and as part of the homework or something, a review of how it’s gone, how you’re feeling, if you felt any kind of satisfaction with it, and if it’s something that you think is worth continuing*” (W3)  “*I think it could be helpful to like see how different goals can join together…like me going to college, like is a step towards achieving like having my own cottage with loads of cats. Like the idea of maybe a goal that you don’t necessarily want to achieve but you need to achieve would be like the first step in achieving something that you do want to achieve*” (W1) |
|  |  | Cognitive components | | - Explore personal understanding of hope - Identify levels of hope in different life domains - Map experiences of hope across life-course and reflect on historical experiences of hope - Opportunity to reflect within (and after) intervention on ways to maintain hope - Reflect on strengths to build self-esteem - Identify values - Participants ranked identifying values, using surveys to quantify hope in different life domains, mapping sources of hope, completing a hope timeline and recalling a hopeful memory as of priority importance | | "*I do think the idea of hope being more personal is more helpful than like finding hope and exampling what it is from, like defining hope for you and what it is for you, and like a lot more helpful*” (W1)  “…*the questionnaire about exploring more and else hopeful areas, I think that provides quite a good basis for what person might want to work on*” (W3)  “…*a good idea for the timeline…you have a different view of everything when you see it all in front of you*” (W6)  “…*it could be quite helpful to maybe identify things that might make a person struggle to feel hopeful, and why. Like if there’s specific parts of their life, or their past experiences that have made them feel less hopeful or, and how about like in what ways that could be addressed from them?*” (W3)  “…*helping you with ways that you can maintain your hope and that, so that even if it's like tougher than you thought you can still like go back to that module and just remember*” (W6)  “…*suppose if it was something addressed in the intervention itself, that could be quite a good place to maybe work on self-esteem, so like if it’s about the person maybe identifying more positives*” (W3)  “*I like the idea of identifying your values*” (W3) |
|  |  | Interpersonal components | | - Exploring characteristics of hopeful relationships within a support network, with an option to discuss relationships generally for people who do not want not want to map their own social networks - Identifying gaps in social support for hope, and planning how to fill - Opportunities for involving family and friends - Participants ranked identifying gaps in their social network related to specific goals, mapping their hopeful relationships, and learning about how relationships affect hope as priorities | | “…*if everyone in your life wasn’t like the nicest to you, and you didn’t really have anyone who was like there for you, I think that* [social network mapping] *would be quite difficult for someone, and wouldn’t necessarily make them feel better*” (W1)  “…*in terms of identifying social networks and the gaps as well, I guess maybe it should focus more on maybe the latter part or the two together so it’s not about what you have at the moment it’s just about what, where you can work and what you think is doable*” (W3)  "*Explaining things to people isn’t like the easiest thing so…a worksheet you could bring home with you with like a vague overview of things that you did…if you think it would be helpful to give to someone in your life so that they could help you with this.*" (W1) |
|  |  | Non-specific factors | | - Supporter offers encouragement - Supporter takes collaborative approach - Supporter is non-judgemental, respectful and validating - Supporter uses active listening - Interpersonal connection with supporter - Participants ranked as highly important discussing, at intervention outset, an overview of the intervention and how the supporter and young person would best work together | | “...*encouragement is like - it just gives you a boost of confidence and I think that's quite important going through the stages to feel within yourself that you're doing okay*” (W6)  “*Something that I find useful is the like person I’m doing it with also doing the task for themselves, because like if I don’t understand something, rather than just explaining it, I find it useful to like look at an example*” (W1)  “…*if they brought up things or started a conversation with them and they say that oh, they don’t want to talk about things, or they don’t want to have that mentioned, then like the people can just take that in and not bring it up or anything*” (W4)  “*I think like emphasis on like understanding and actually listening. Because I think a lot of like interventions and that kind of thing are a bit more like clinical, and “We can help fix your problems,” and like that kind of stuff, rather than “We can listen to your problems”*” (W1)  "…[if] *I had a miserable time and I hated it, I don’t need you to be like, “Oh you did it, you succeeded,” I find that like a little bit like annoying*” (W1)  “…*create obviously a bond with someone to be able to like talk to them*” (W4) |
|  |  | Additional components | | - Support EET activity, at personal preference - Mental health/mental health problem focused components, at personal preference | | "*Any sort of mental health sort of things for people that really struggle…there’s not enough help*" (W4) |
|  | HOW | One-to-one intervention, with supported and self-directed components | | - Primarily one-to-one intervention, with option for group sessions at individual preference - Mixture of supported and self-directed activities | | “*I think the idea of it being one-to-one, like I’ve done lots of anxiety stuff in groups, but say there’s…four or us saying, “This is something I really struggle with,” so you’re spending time talking about that where I can be sitting there thinking, ‘This is not something I remotely struggle with’*” (W1)  “…*if like some of the people wanted to do it like on their own like one-to-one with people, or like if they’re happy to do it in groups or, I think maybe it would be good to have like that option there*” (W4)  “*I guess maybe in terms of if this is a person who like they’re familiar with, they might not necessarily have the time to always be with the person doing it. So, I suppose if they’re able to sort of do self-initiated work, maybe a balance between the two*” (W3) |
|  | WHERE | Setting | | - Primarily in-person, with option for flexible online delivery as individually preferred - Non-clinical setting - ‘Active’ delivery, encouraging taking the intervention outside of the home/community venue - Connection with mental health services for signposting and onward referral, where relevant | | “…*places like coffee shops…or even just outside, I think like we’d be doing a semi-structured like a work but then obviously sitting down in some sort of open spaces*” (W3)  “…*putting someone in like a neutral zone, and also like if you do feel stressed you can like go and look at something, rather than being forced to sit down and talk about this for like hours, or like if you’re like uncomfortable you can like take a second to go like look at the pretty flowers*” (W1)  “…*whoever’s running the session, if they’re able to maybe reach out to different mental health services...be able to link people to it and help recommend a service if they think it could be beneficial”* (W3) |
|  | WHEN and HOW MUCH | Session number and scheduling | | - Six modules across a flexible number of sessions - Flexible session timing, duration, intensity, and pacing, as individually preferred - Potential for long-term support - Provision of activities for use beyond end of intervention | | “*I think as like a starting point of this is what we’re presenting to people I think six feels like a good number*” (W1)  “*I think the modules are a good idea and I like the fact that you can take as much time as you want on each of them, because everyone learns at like a different pace*” (W6)  “[If sessions were only offered] *really early in the morning, that I find incredibly difficult, so any positive help that they could have, is kind of also you have to take into consideration me getting out of bed really early when I’m like exhausted*” (W1)  "…*if other people have like other things going where it couldn’t be maybe as long, or they wanted to make it shorter, then that could happen and that would be good and helpful towards them people*" (W4)  "…*something you can carry forward beyond this*” (W4) |
|  | TAILORING | High level of flexibility | | - Early discussion to establish problems of personal relevance - Modules, and activities within modules, should not be mandatory - Homework is helpful, but should not be mandatory - Opportunities to adapt, review, revise and repeat learning and activities - Activities completable in different ways (e.g. written, audio, visual/arts-based), as individually preferred | | “*I think the idea of like talking to someone about* [what to focus on in the intervention] *is more helpful. I think properly like figuring out what they could actually use help with*” (W1)  “…*regular check-ins about how it’s going and then potentially changing some of the later steps or having the ability to reflect on that and make adjustments*" (W3)”  “*I think it’s quite good that it’s giving the option to repeat certain activities, especially towards the end of the modules, so you might be looking at it from different perspectives, having completed all of the modules*…[also] *it probably makes it quite a bit more accessible for maybe people potentially having different disabilities*” (W1)  “…*something useful that you can do and think about in between to sort of keep all of the learning sort of going*” (W6)  “[Homework] *being an option but not something that there’s load of pressure on to do*" (W4)  “*I think it could be helpful in that* [final] *session maybe having an overview of the other sessions so sort of reminding people of what they might have taken from the other sessions*” (W3)  “…*the different options for doing the different activities, I think that's quite useful because some people are academic, but some people are more creative. So, I think it's quite good to acknowledge like the different ways people learn*” (W6) |
| iii. Who are possible non-specialists that could credibly deliver the intervention? | WHO PROVIDES | Provider background | | - Youth worker with some mental health expertise, but not a clinical/medical professional, and lived experience of being NEET | | “*I think someone like a youth worker would be helpful in that sort of situation, someone like I suppose it would be helpful for it to be a bit less clinical…people also with more like lived experience and maybe not looking at it from quite a clinical perspective, but more from personal”* (W3)  *“…some general knowledge of mental health would be helpful for the person who’s supporting… there probably is quite a lot of lack of understanding and stigma around it*" (W1) |
|  |  | Youth-initiated mentor model | | - Benefit of youth-initiated mentor, with provision of a mentor from a community organisation if not possible/wanted | | “…*I think it's quite good because some people find it hard to like keep meeting new people, you know when you go through like different things to help you get back on track, you meet quite a few people for a short period of time. But if you have someone that's just kind of like there long-term it can make you feel more comfortable, and you can open up a lot more*" (W6)  “…*having someone that maybe they already trust, with that kind of situation… only worry that I would have had with it, is for people who perhaps don’t have many connections and might not have someone to go to…personally I can think of people that I would perhaps like, so in my community team as I’m under like a mental health service, I have a key-worker and I can imagine doing this kind of work with her. I was thinking possibly also a parent like, my mum or someone similar like a parental kind of figure*" (W3) |
|  |  | Training | | - Emphasis on personal meaning focus of intervention - Emphasis on importance of confidentiality, especially for youth-initiated mentors | | “…*the emphasis that it’s not about making the person succeed in life but emphasising their happiness and wellbeing*" (W3)  “…*making sure that you’re not biased so that like if someone says something and you think that ‘That’s ridiculous’, but that could be like very common in their life*” (W1)  “*I think it would be important to emphasise on like the confidentiality aspect of it, because if you like told someone like an issue that you had and then like something related to that issue came up in like an argument and someone you knew, you wouldn’t, you need to like understand that that’s not appropriate to tell that person*” (W1) |
| ii. What are the most meaningful short- and long-term outcomes related to hope and its theorised effects on mental health and social functioning, and how can these be measured? | | | Short, medium, and long-term outcomes | | - Increased hope as the most realistic and important short-term outcome - Mental health is very important, but should not necessarily be the primary outcome - Aspirations, activity and social outcomes are important secondary outcomes - Psychological outcomes should include flexibility and open-mindedness - Participants ranked the following outcomes as priorities; general hope, wellbeing, help-seeking, hope relating to EET domains, social relationships, EET and broader meaningful activities | “…*the intervention itself, it’s probably easier to make progress* [with hope] *within the short term*" (W3)  “*I do think that* [improved mental health as a primary outcome] *would be like a lot of pressure as a thing that like you can very easily fail at*” (W1)  "…*emphasising the importance of… meaningful activity and social networks*" (W3)  “*Just like inspiration for the person, would want to go back into like that every day, kind of education or wanting to find a job or even just volunteering or something*” (W6)  “…*maybe within the psychological part, maybe flexibility and open-mindedness*” (W3) |
