# Supplementary material for: Developing a hope-focused intervention to prevent mental health problems and improve social outcomes for young women who are not in education, employment, or training (NEET): A qualitative co-design study in deprived coastal communities in South-East England
Source: PLoS One. 2024 May 31;19(5):e0304470. doi: 10.1371/journal.pone.0304470 (PMC11142577; doi:10.1371/journal.pone.0304470)
Supplement: S1 Fig — Module names are presented with module number in parentheses. Primary axis presents percentage of participants ranking each module 1 (most important) to 6 (least important). Secondary axis presents mean rank. (DOCX) [file pone.0304470.s003.docx]

**S1 Fig. Intervention module importance rankings by Phase 2 co-design participants**. Module names are presented with module number in parentheses. Primary axis presents percentage of participants ranking each module 1 (most important) to 6 (least important). Secondary axis presents mean rank.


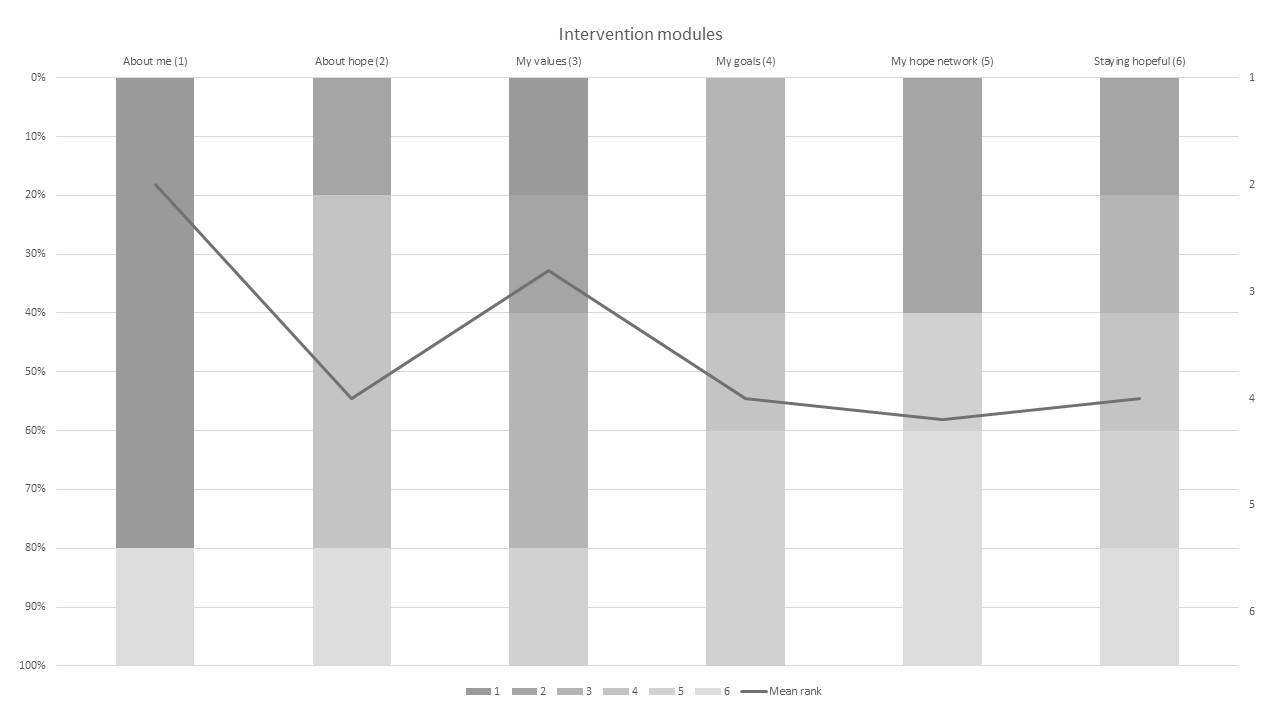


*Notes: Module names are presented with module number in parentheses. Primary axis presents percentage of participants ranking each module 1 (most important) to 6 (least important). Secondary axis presents mean rank.*
