# Supplementary material for: Developing a hope-focused intervention to prevent mental health problems and improve social outcomes for young women who are not in education, employment, or training (NEET): A qualitative co-design study in deprived coastal communities in South-East England
Source: PLoS One. 2024 May 31;19(5):e0304470. doi: 10.1371/journal.pone.0304470 (PMC11142577; doi:10.1371/journal.pone.0304470)
Supplement: S2 Fig — Module name and number presented as figure titles. Primary axes present percentage of participants ranking each module activity 1 (most important) to 4 (least important). Secondary axes present mean rank. (DOCX) [file pone.0304470.s004.docx]

**S2 Fig. Intervention module activities importance rankings by Phase 2 co-design participants.** Module name and number presented as figure titles. Primary axes present percentage of participants ranking each module activity 1 (most important) to 4 (least important). Secondary axes present mean rank.


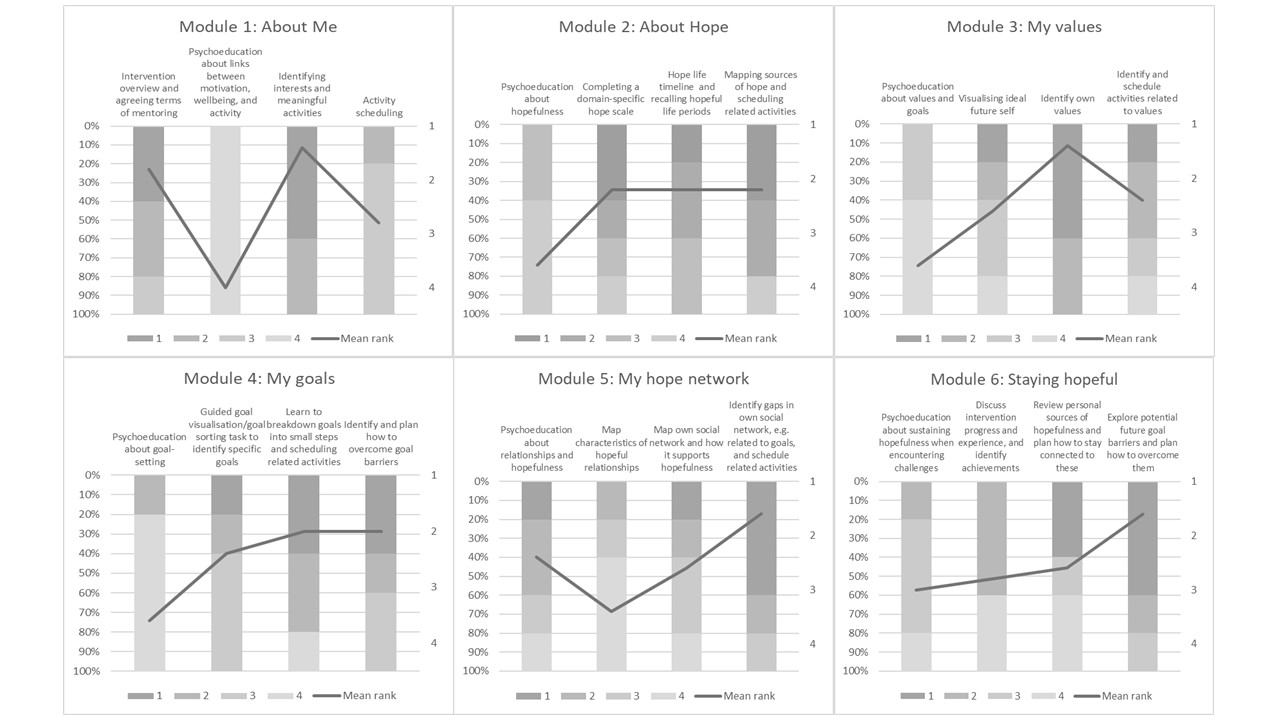


*Notes: Module name and number presented as figure titles. Primary axes present percentage of participants ranking each module activity 1 (most important) to 4 (least important). Secondary axes present mean rank.*
