# Supplementary material for: Developing a hope-focused intervention to prevent mental health problems and improve social outcomes for young women who are not in education, employment, or training (NEET): A qualitative co-design study in deprived coastal communities in South-East England
Source: PLoS One. 2024 May 31;19(5):e0304470. doi: 10.1371/journal.pone.0304470 (PMC11142577; doi:10.1371/journal.pone.0304470)
Supplement: S3 Fig — Primary axis presents percentage of participants ranking each outcome 1 (most important) to 10 (least important). Secondary axis presents mean ranks. (DOCX) [file pone.0304470.s005.docx]

**S3 Fig. Intervention outcomes importance rankings by Phase 2 co-design participants.** Primary axis presents percentage of participants ranking each outcome 1 (most important) to 10 (least important). Secondary axis presents mean ranks.


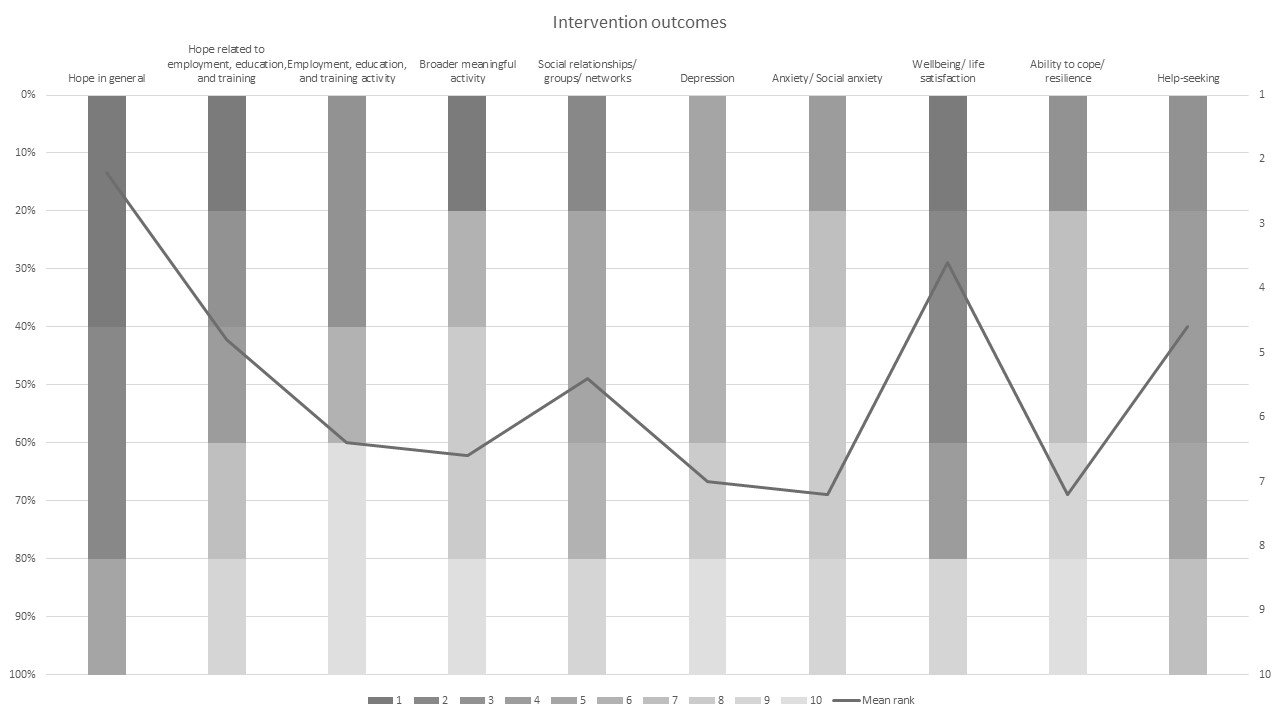


*Notes: Primary axis presents percentage of participants ranking each outcome 1 (most important) to 10 (least important). Secondary axis presents mean ranks.*
